# Sniper: improved SNP discovery by multiply mapping deep sequenced reads

**DOI:** 10.1186/gb-2011-12-6-r55

**Published:** 2011-06-20

**Authors:** Daniel F Simola, Junhyong Kim

**Affiliations:** 1 Department of Biology, University of Pennsylvania, 433 S. University Ave, Philadelphia, PA 19104, USA; 2 Department of Cell and Developmental Biology, University of Pennsylvania, 421 Curie Blvd, Philadelphia, PA 19104, USA; 3 Penn Genome Frontiers Institute, University of Pennsylvania, 433 S. University Ave, Philadelphia, PA 19104, USA

## Abstract

SNP (single nucleotide polymorphism) discovery using next-generation sequencing data remains difficult primarily because of redundant genomic regions, such as interspersed repetitive elements and paralogous genes, present in all eukaryotic genomes. To address this problem, we developed Sniper, a novel multi-locus Bayesian probabilistic model and a computationally efficient algorithm that explicitly incorporates sequence reads that map to multiple genomic loci. Our model fully accounts for sequencing error, template bias, and multi-locus SNP combinations, maintaining high sensitivity and specificity under a broad range of conditions. An implementation of Sniper is freely available at http://kim.bio.upenn.edu/software/sniper.shtml.

## Background

The advent of next-generation, short-read sequencing (NGS) technologies has enabled large-scale, whole-genome resequencing studies that aim to discover novel SNPs and other population genetic variations. Perhaps the most ambitious of these studies involves sequencing over 1,000 individual human genomes in order to map human genetic variation at a fine scale and to support genome-wide phenotypic and disease association studies [1]. Previous genome resequencing efforts have developed a variety of approaches to identify SNPs, including straightforward decision rules such as minimum coverage and quality cutoffs along with filters that mask reads aligning to repetitive genomic templates [2]; Bayesian algorithms that explicitly model sequencing chemistry and take full advantage of read-specific quality scores [3,4]; unsupervised [5] and supervised [6,7] machine-learning algorithms trained to distinguish sequencing errors from SNPs; and an alignment method that performs read mapping using all four nucleotide probabilities per-locus instead of the most probable call [8]. Although these tools have successfully predicted many novel SNPs, genomes themselves contain inherent degeneracy due to redundant paralogous sequences and low complexity repetitive elements, while NGS data exhibit non-negligible sequencing errors and severe biases in sequencing coverage generated during genomic DNA library preparation [9]. These practical issues make it difficult, even with sophisticated procedures, to discover true SNPs accurately without concurrently predicting many false SNPs. A recent study evaluated the performance of three NGS technologies (Illumina, ABI Solid, 454) using near-saturating sequence coverage (approximately 188×) over the same genomic sequence, reporting false positive and false negative SNP discovery rates of 3 to 12% and 1 to 8%, respectively [9]. While these error rates may seem manageable, they were estimated using SNPs occurring in high complexity (that is, unique) regions of the human genome and fail to account for errors resulting from repetitive or degenerate genomic sequence. Our investigations reported below suggest that the standard methods of SNP discovery will have much higher error rates when accounting for degenerate sequence. Furthermore, even moderate error rates can severely affect the use of SNPs in genome-wide association studies. As a simple example, suppose we have a genome-wide association study with 1,000 cases and 1,000 controls, a SNP segregating at 5% frequency, and a simple contrast of homozygous reference genotype versus heterozygous SNP genotype. Genome-wide significance can be obtained if the case:control SNP frequency ratio is approximately 3:1. With a 8% false negative rate and a 12% false positive rate, we need approximately 40% larger sample size for both cases and controls to obtain the same genome-wide significance.

**Figure 1:**
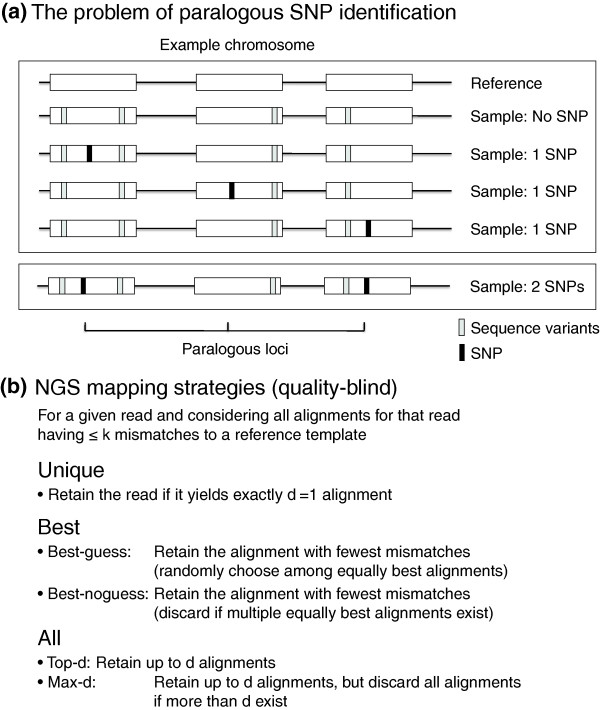
**Multi-locus model for SNP discovery using multiply mapped reads**. **(a) **Distinct SNPs occurring in paralogous genomic regions are largely undetectable using either unique or best-guess read mapping because sequence reads containing a SNP can map to multiple paralogous loci with equal confidence. The figure shows an example with three paralogous loci and possible combinations for the multi-locus genome for up to one SNP in the exact paralogous loci (cases labeled 'No SNP' and '1 SNP'). Sequences in positions other than the exact paralogous loci may have other variations (shown as gray bars). Higher order configurations are possible, as shown in the bottom '2 SNP' case. However, such configurations can only arise through exact parallel mutation or segregating SNPs prior to genomic duplication. **(b) **Overview of read mapping strategies employed in this study. Quality-blind means that per-base quality estimates (probability that a base call is correct) are not considered during the mapping process.

Although weaknesses associated with current sequencing technologies *per se *may be mitigated by future improvements that yield sufficiently longer reads or improved genomic coverage or sequencing error rate, the genome sequences of most organisms contain inherent redundancies that will nevertheless impede variant detection. A variety of sequence elements are repeated with high similarity and occur at various length scales, including simple sequence repeats (< 0.1 kb) [10], Alus (0.3 kb), DNA transposons (1 to 2 kb), non-long terminal repeat (4 to 6 kb) and long terminal repeat (< 10 kb) transposons [11], duplicated protein domains (< 0.5 kb) [12], and paralogous and duplicated open reading frames (> 1 kb) [13]. In particular, many repetitive elements remain actively mobile within the human genome, contribute to human genomic diversity [14,15], and have been implicated in various human diseases [16]. Therefore, SNP discovery in redundant sequence contexts - beyond just a proof of principle - is relevant for genome-wide association studies. Detecting true SNPs in these degenerate sequence contexts is difficult because an individual sequence read can show significant alignment to multiple genomic locations, making it difficult to distinguish between a SNP and a base-call error (Figure 1a). Although a SNP occurring within a repetitive sequence may be identified from overlapping reads that are anchored by unique flanking template, accurate mapping may be impossible if the length of the repetitive sequence is greater than the length of the read. For this reason previous SNP identification methods have utilized only the subset of sequenced reads that can be mapped uniquely to a reference genome [1,3-5]. A standard, quality-blind definition of uniqueness allows few sequence mismatches between a read and its potential genomic template (typically ≤3 mismatches, but the exact cutoff is a function of expected heterozygosity and sequencing error), discarding a read that has more than one such alignment (Figure 1b). Thus, focusing solely on uniquely mapped reads commonly results in discarding a significant percentage of otherwise mappable reads. Discarding reads especially disproportionately affects low complexity regions such as centromeric or telomeric regions, which end up with much lower coverage than expected from the average coverage. Consequently, SNPs occurring in redundant sequence contexts may be missed.

In our results below, we first investigate the effect of redundant sequences on unique read coverage based on current NGS parameters. Exclusive use of uniquely mapped reads can significantly reduce information for SNP discovery (increasing the chance of false negative or incorrectly genotyped SNPs) and can, in some cases, create spurious map locations (increasing the chance of false positive SNPs). We then propose a novel SNP discovery algorithm that uses information from all mappable reads, including non-unique (multiply mapped) reads. Our method significantly improves the false SNP discovery error rate while maximizing prediction accuracy by utilizing a complete read map that includes both singly mapped and multiply mapped reads together with a Bayesian probabilistic model that explicitly accounts for multi-locus variation arising from multiply mapped reads. We describe this model below and provide a computationally efficient algorithm that prevents an explosion in the size of a read map via an approximation strategy. We validated our algorithm's performance on an ABI Sanger verified portion of the human genome using Illumina 1G sequencing data; on a simulation test suite derived from concatenations of yeast ribosomal protein loci; and on a single high-coverage (trio) individual from the 1000 Genomes Project.

## Results

### Redundancy structure of the human genome

**Figure 2:**
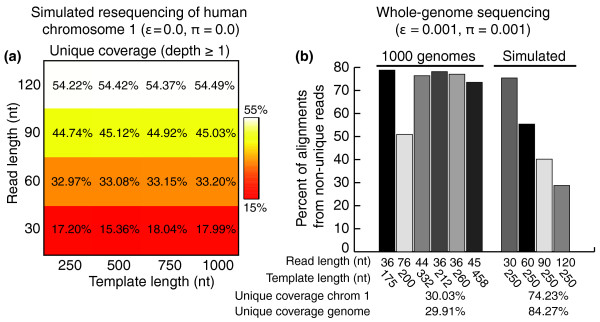
**Redundancy structure analysis of the human genome**. **(a) **We generated 2 × 10^6 ^PE reads randomly and with perfect fidelity (error rate e = 0; divergence p = 0) from the NCBI *Homo sapiens*genome. Read sets were generated for four different fragment lengths (250, 500, 750, and 1,000 nucleotides) and four different sequencing read lengths (30, 60, 90, and 120 nucleotides) and mapped to their respective genome using Bowtie, allowing 1, 2, 3, or 3 alignment mismatches depending on read length. Heat map cells represent the percent of sampled reads having more than one valid alignment, averaged over the total number of reads for that data set. The distributions of the number of alignments per read are shown in Additional file 2. **(b) **Six experimental and four simulated read sets containing both base-call sequencing errors (e ≈ 0.001) and population sequence divergence (p ≈ 0.001) were mapped to the reference human genome. The percent of non-unique alignments is shown for each read set. Percent of loci covered by at least one unique alignment was computed after pooling all experimental or simulated read maps. Nt, nucleotide.

To evaluate the extent to which genomic sequence redundancy affects read mapping, we first analyzed the redundancy structure of the human genome by randomly sampling paired-end (PE) reads from the reference sequence and estimating the fraction of reads exhibiting only one versus multiple valid alignments (read multiplicity), using chromosome 1 as a representative sample. We defined a read to be unique if it aligns to exactly one location in the genome allowing at most *k *mismatches. We simulated 16 sequencing experiments by generating independent sets of 25 × 10^6 ^PE reads without addition of base-call errors or sequence divergence (that is, perfect resequencing); read sets were simulated for four read lengths (30, 60, 90, and 120 nucleotides) and four template (fragment) lengths (means of 250, 500, 750, and 1,000 nucleotides), yielding 0.47, 0.94, 1.41, and 1.88-fold coverage genome-wide, respectively. Assuming a constant base-call sequencing error rate across the length of a read, we set the number of allowable mismatches to be proportional to read length, starting with a 1-mismatch cutoff for 30-mer reads and ending with a 3-mismatch cutoff for 120-mer reads. (Reads of 60 and 90 nucleotides are aligned with 2 and 3 mismatches, respectively.) Following PE alignment, we also aligned unmapped reads as single-end (SE) reads, resulting in a conservative estimate of uniqueness. From 15 to 55% of nucleotide loci on chromosome 1 (average 37.4%) are covered uniquely in any experiment, with longer read lengths yielding greater coverage, as expected (Figure 2a). This relatively low coverage suggests that a large portion of the human genome is composed of low complexity sequences that are highly degenerate by edit distance. Interestingly, template length appears to have negligible effect on unique coverage, suggesting that the distribution of redundant sequence elements extends across a range of length scales (see Text S1 in Additional file 1). Since we sampled PE reads without error, all of the remaining non-unique reads must also map to the genome, although via multiple alignments (Additional file 2). Thus, in the context of whole-genome sequencing, uniquely mapped reads show an inherently limited ability to cover the human genome (see also [1,17]). By mapping non-unique reads together with unique reads - retaining only those reads with at most 50 alignments (6.5% of the remaining 17.5% of reads on average) - 17 to 37% of the resulting read map (average 24.5%) correspond to non-unique reads. Including these redundant alignments yields a moderate 3.5 to 8.3% (average 4.8%) increase in the number of covered genomic loci and a substantial 26.4 to 45.3% (average 38%) increase in read depth per locus compared to the unique read map (Additional file 3). This suggests that while the majority of loci are covered by at least one unique subsequence, using unique reads only may bias genotyping over even mildly degenerate loci. Thus, significant improvement in both number of loci covered by at least one read and read depth per locus can be achieved by including just a portion of the entire non-unique read map.

Our estimates of the impact of non-unique reads are likely conservative: the above results do not account for population-level divergence, nor do they explicitly account for base-call errors. To evaluate the extent of non-unique mapping in the presence of sequencing error and genomic divergence, we mapped 250 × 10^6 ^PE reads taken from the 1000 Genomes Project as well as 181 × 10^6 ^PE reads sampled from the reference human genome at different read and template lengths; this corresponds to 7.5-fold and 4.7-fold expected genome-wide coverage for experimental and simulated read sets, respectively. For simulated reads we randomly introduced base-call sequencing errors uniformly using a 0.1% error rate. We mapped reads to the reference genome allowing up to 2 mismatches and 200 valid alignments per read. Under these conditions, a substantial percentage of alignments correspond to non-unique reads for all read maps, averaging 50% for simulated and 72% for experimental maps (Figure 2b). Pooling all experimental or simulated reads, the percentage of the human genome covered solely by unique reads is 29.91% and 84.27%, respectively (30.03% and 74.23% for chromosome 1). Thus, we estimate that approximately 15% of the human genome cannot be covered by unique reads alone using current NGS parameters (similar to estimates reported in [1]). As expected, endogenous repetitive elements, including functionally relevant elements, are important determinants of this redundant portion of the human genome (Text S1 in Additional file 1). We note that the discrepancy between Figures 2a and 2b for simulated maps (approximately 55% versus approximately 74%) is explained first by the lower coverage in Figure 2a and second by the difference in mismatches (three versus two, respectively); there is a negative correlation between mismatches and unique coverage, so using two mismatches should yield more mapped reads than three mismatches (in the presence of sequencing error). The estimates of unique mapping in Figure 2b thus may be conservative for very long reads where 3' ends show a rapidly increasing base-call error rate. In addition, experimental data map 36% fewer unique reads than synthetic reads, indicating the presence of more extensive genomic differences or elevated error rates among experimental NGS data sets. While our estimate of the redundant portion of the human genome is based on PE reads, many existing data sets utilize SE reads with shorter read lengths; these data can yield severely reduced unique mapping. For example, in our 1000 Genomes analysis above, 48.2% of the uniquely mapped reads only map as singletons rather than PE reads. Also, analysis of a *Saccharomyces cerevisiae *genome sequenced using 36-nucleotide Illumina 1G PE reads revealed that 52.0 to 65.5% of reads map non-uniquely when treated as singletons compared to 8.2 to 9.3% when treated as paired (depending on mismatches; data not shown). Thus, when considering NGS data sets containing genomic changes, using a unique read map impedes discovery of novel polymorphisms for a significant portion of any genome, while non-unique alignments become increasingly prevalent.

### Extent of spurious alignments due to mapping strategy

Given the prevalence of singleton mapping among PE data sets (as described above), we wondered whether SE reads show reduced coverage over SNP loci in particular, and whether they tend to be mapped to the wrong genomic location. For example, a read could be mapped incorrectly if it corresponds to a region that is redundant and by chance shows more similarity to an incorrect paralogous region. Such spurious read mapping may occur if the sequenced read has either base-call errors or true polymorphisms (Additional file 4). While this scenario applies to unique mapping as described above, it may be of particular relevance when employing a greedy approximation strategy to utilize additional reads that are not strictly unique (for example, the 'best' strategies in Figure 1b). With these approximations, although multiple valid alignments may exist for a read, the one containing the fewest mismatches is selected as the correct alignment.

We evaluated the occurrence of discarded and spuriously mapped reads in the presence of true genomic divergence by considering four scenarios: (1) error-free sequencing and unique mapping; (2) error-free sequencing and best-guess mapping; (3) error-prone sequencing and unique mapping; (4) error-prone sequencing and best-guess mapping. Note that the error-free scenarios are equivalent to error-prone scenarios without sequence divergence, which could affect confidence in a SNP call at a false locus. Also, we did not evaluate the best no-guess strategy here since it essentially behaves like the unique strategy when error and divergence rates are low, as in the following analysis. Using human chromosome 1 as a reference, we randomly introduced 1% polymorphic divergence and extracted all read strings that overlap every SNP locus, for 30, 60, 90, and 120-nucleotide read lengths. For error-prone scenarios we randomly introduced 1% base-call errors uniformly over read strings. In this manner we generated five independent replicate read sets for each read length. We then aligned these read strings back to the entire human genome allowing two mismatches and counted the average number of reads that map (1) uniquely to the corresponding SNP locus, (2) to the SNP locus with fewest mismatches (best-guess), (3) somewhere else in the genome. Overall, we found negligible false alignments with the unique map, with or without sequencing error (Table 1). However, the best-guess map yielded 12.1% false alignments for error-free reads and 13.0% false alignments for error-prone reads with 30-nucleotide reads; longer reads showed up to 3.5% false mapping. This suggests that the potential for false mapping is largely restricted to 'best' strategies and shorter read lengths, whereas unique mapping almost always yields correct alignments (at least over true SNP loci). Thus, spurious mapping becomes most relevant when short, SE reads are used extensively, such as for low-coverage 1000 Genomes Project data (as well as ChIP-seq and RNA-seq data).

**Table 1:** Extent of spurious alignment due to unique or best-guess mapping strategies

Condition	Read length	SNP reads	Total aligned	Discarded	Misaligned	Misaligned/Aligned
Best, no error	30	7,413,324	99.96%	0.04%	12.11%	12.11%
	60	14,826,840	99.81%	0.19%	3.02%	3.02%
	90	22,240,926	99.55%	0.45%	1.15%	1.15%
	120	29,654,088	99.21%	0.79%	0.66%	0.67%
Uni, no error	30	7,413,324	74.94%	25.06%	0.00%	0.00%
	60	14,826,840	89.20%	10.80%	0.00%	0.00%
	90	22,240,836	93.74%	6.26%	0.00%	0.00%
	120	29,654,088	94.98%	5.02%	0.00%	0.00%
Best, 1% error	30	4,388,175	96.40%	3.60%	12.53%	13.00%
	60	2,771,304	86.33%	13.67%	2.99%	3.46%
	90	2,252,588	74.20%	20.62%	1.02%	1.38%
	120	2,132,976	62.14%	37.86%	0.51%	0.82%
Uni, 1% error	30	4,388,175	72.98%	27.02%	0.07%	0.09%
	60	2,771,304	78.01%	21.99%	0.06%	0.08%
	90	2,252,588	70.34%	29.66%	0.03%	0.05%
	120	2,132,976	59.82%	40.18%	0.02%	0.03%

Mapping of reads that overlap synthetic SNPs on human chromosome 1 was performed allowing up to *k *= 2 mismatches per read under four mapping conditions (best-guess without base-call error; best-guess with 1% base-call error; unique without error; unique with 1% base-call error) and four read lengths (30, 60, 90, and 120 nucleotides). Total mapped SNP reads and percentages of aligned, discarded, misaligned, and the ratio of misaligned to aligned reads are shown for each experiment. A read is considered misaligned if it does not overlap the SNP locus used to generate the read. See Figure 1b for a description of mapping strategies.

We also counted reads that overlap a SNP locus and are discarded because no valid map location could be identified (because these reads would have more than *k *bases that differ from any location in the reference genome). This is an important consideration for many recent long read NGS data sets, since longer reads tend to have more base-call errors than short reads. For example, for a 1% base-call error rate, one mismatch mapping, and no sequence divergence, this is expected to occur for approximately 3.6% of 30-nucleotide reads, 12.1% of 60-nucleotide reads, 22.7% of 90-nucleotide reads, and 33.8% of 120-nucleotide reads. Using our SNP-derived read sets and two mismatches, we found that 5 to 25% of error-free and 22 to 40% of error-prone reads are discarded for unique mapping, and 0 to 1% of error-free and 4 to 38% of error-prone reads are discarded for best-guess mapping. Using only one mismatch exacerbates read loss: 3 to 21% of error-free and 25 to 74% of error-prone reads that overlap SNP loci are discarded (Additional file 5).

Thus, when using mapping conditions that are currently standard for SNP discovery in the human genome (strictly or approximately unique mapping using two mismatches), we find that a significant number of reads that overlap true SNP loci are discarded during mapping - regardless of sequencing errors and read length - because they have more differences than allowed compared to the reference genome. Moreover, false mapping appears to be a relevant concern for best-guess strategies; although the extent of false mapping decreases substantially for longer reads, just a few spurious alignments may be sufficient to add significant bias to SNP calls (as implied by our results below). Alternatively, a multiple read mapping strategy will recover many of the reads discarded by strictly unique mapping while avoiding the possibility of spurious alignment. (While reads discarded by best-guess mapping cannot be recovered, this strategy may tolerate more mismatches.) Based on these considerations, we developed a Bayesian model for SNP discovery that integrates the joint information from multiply mapped genomic locations for any given read.

### Overview of the Bayesian genotyping model

A full probabilistic model for SNP discovery can be specified in a straightforward manner, but a model that incorporates the full joint probability of genotypes at all loci and all possible sequencing reads would be computationally prohibitive. Here, we employ three assumptions to manage this complexity: (1) the sequencing chemistry is sufficiently accurate that the probability of a given sequenced read being generated from a genomic template that is very different from the read string is negligible; (2) the resequenced genome is sufficiently similar to the reference genome such that the alignment of the read strings to the reference genome accurately delineates the possible genomic positions of the resequenced genome; and (3) that for any multiply mapped reads, at most only one of the possible mapped loci have sequence deviation from the reference genome at the exact same position within the sequenced read string. Assumptions 1 and 2 are standard to current short read mapping analyses that employ *k*-bounded approaches to map sequenced reads. We assume that the user chooses the bound *k*, based on prior ideas about the resequenced genome. (Note that all read map algorithms have such a bound as a user-selected parameter.) Assumption 3 restricts the joint combination of possible genomic variants in the resequenced genome. Figure 1a shows an example of three paralogous loci that may be the template for a given sequence read, where black bars show a possible SNP genotype of interest while gray bars show sequence variation around the loci. The multi-locus genomic genotypes for the SNP locus may be identical to the reference genome (labeled 'No SNP') or have a SNP in one of three possible paralogous loci (labeled '1 SNP'). Our model allows for all 'No SNP' or '1 SNP' multi-locus combinations. The bottom example in Figure 1a shows an example of a highly unlikely '2 SNP' combination. Identical SNPs in the same paralogous loci can only arise if there were exact parallel mutations or if a single SNP arose in an ancestral genome before the multiplication of the paralogous loci. Both of these events have very low probability: the probability of exact parallel mutations that segregate at appreciable frequency or the probability that a SNP in an ancestral genome that is different from the reference genome is segregating at both copies of descendent paralogs would both be close to zero.

A detailed elaboration of the probability model is given in Text S2 in Additional file 1. Here we outline the main features of the model. We first define:(1)

as the probability of observing the read string *r_i _*given that the particular genomic substring *s *of reference genome *G *served as the sequencing template; therefore, *P*(*r_i_|s,G) *is determined by the fidelity of the sequencing chemistry. This probability can be modeled based on known error rates of the sequencing platform or by the quality (phred) scores for individual sequenced nucleotides. The template string *s *may correspond to multiple locations in the genome with sufficient similarity to the read string. Thus, we model the probability:(2)

which is the probability that a particular substring *s *of genome *G *became the template for the read string *r_i_*. This probability is determined by the process of template preparation and may be affected by biases of the library preparation chemistry. The probability models Equation 1 and Equation 2 can be put together to compute the probability:(3)

where *d *is the number of possible template strings for read *r_i_*. Assuming independence of read strings *r_i _*given the genome and applying the Bayesian formula yields:(4)

where *G_xy _*denotes one possible genome with the genotype *xy *at a locus of interest, and where the denominator is a summation over all possible genotypes *XY*. The term *P*(*G_xy_*) is the prior probability of the genome and can be modeled, for example, from the population-level expected heterozygosity. Finally, since many different genomic configurations (whole-genome sequences) can have the same genotype *xy *at any particular locus, the marginal probability of the genotype at *xy *at locus *g_l _*is:(5)

where *C*(*G_xy_*) denotes the possible configurations of the unknown genome *G*, fixing genotype *xy *at locus *g_l_*.

The possible configurations can be enumerated from assumptions about how different the sampled genome *G *is compared to the reference genome (Text S2 in Additional file 1). In practice, by assumptions 1 to 3 noted above, the possible genomic configurations are restricted to loci that are homologous to read strings (within *k*-bounded differences from the reference sequence) and having at most one SNP within the multiply mapped positions (as shown below and in Figure 1a).

Finally, using our Bayesian scheme, we can set a threshold that defines the minimum posterior probability to accept a SNP as significantly different from the reference genome. This threshold can be interpreted as a stringency parameter reflecting minimum confidence in a set of genotypes.

### Model analysis

The posterior probability of a given genotype is determined by the likelihood of the reads given the prior probability (the numerator of Equation 4), normalized by the total marginal probability of the reads (the denominator of Equation 4). The main component of the numerator determining the posterior probability is the likelihood of the reads. Taking Equation 3 above, the likelihood of any given read is decomposed into two terms (see also Equation 4 in Text S2 in Additional file 1):(6)

where *p_xy _*are the terms whose probability is a function of the genotype *g_l _*at the locus of interest and *p_bg _*represent the 'background' probability of reads contributed from other genomic alignments. Suppose there is an equal probability that any of the *k*-bounded error regions of the genome might have acted as the template for the read string *r_i_*. Then we have:(7)

Here, *d *is the number of alignments to other genomic loci. (Note that we use a slightly different accounting of the number of alignments than used in Text S2 in Additional file 1 to avoid notational complexity from considering diploid states.) From Equation 7, it is immediate that if a read aligns to many genomic loci (*d *is large), then *P*(*r_i_|G*) goes to a constant regardless of the genotype *g_l_*, and each genotype's posterior probability becomes equally probable. Therefore, as expected, if a locus of interest is covered by sequence reads with a large number of possible alignments to other genomic loci, we have no confidence in the genotype of the locus.

The likelihood of the entire read set at a locus of interest is given by , the product of Equation 6 over the aligned reads. Suppose that we have a read depth of *n*, and assume that each of the reads aligns to *d *other genomic loci. For the purposes of analysis only, also assume that the likelihood of each read is an identical function of the genotype, denoted *p_xy_*, regardless of the read itself. The odds ratio of the posterior probability of genotype *xy *versus *x*'*y*' is given as:(8)

where the last term is by Taylor expansion. The log-odds ratio is meaningful (that is, different from 1) if *O*(*n|d*) ≠ 0. Equation 8 intuitively shows that the log-odds ratio becomes different from even-odds in proportion to the read depth *n *and is inversely proportional to the number of alignments per read *d*. Equation 8 suggests that if the number of alignments at a given locus is much larger than the read depth, then we will not have meaningful information about the genotype, and thus such loci should be considered unknown. Consistent with this, we find a clear negative correlation between posterior probability and degeneracy (r = -0.98, *P *< 10^-6^) when plotting the distribution of posterior probabilities for loci whose maximum *a posteriori *(MAP) genotype indicates a SNP, grouped by the ratio *d/n *(Additional file 6).

Expected read depth of a sequencing run can be used as a cutoff for the maximum number of alignments considered at a given locus. This would greatly reduce the computational load for genotyping. In practice, alignment algorithms for next-generation sequencing produce read alignments up to a user-specified mismatch cutoff, denoted *k*. As the cutoff value *k *is increased, the read depth is expected to go up for any given locus as more of the sequencing reads are relevant to any given locus. However, the number of alignments *d *is also an increasing function of *k*. That is, we expect , where *α *and *β *are increasing functions of *k*. The exact behavior of *α *and *β *are genome-dependent. Therefore, one strategy would be to apply an alignment algorithm at various values of *k *and chose a resulting read map that maximizes the ratio of average read depth versus average number of alignments. Additional file 7 shows this ratio for various alignment mismatch values of *k *and datasets used in this paper.

### Multiply mapped reads improve accuracy and false discovery rate

To validate the performance of our method, called Sniper (SNP Identification using the Probability of Every Read), we estimated genotypes for 261 kb of the human genome for 4 individuals using 36-nucleotide Illumina 1G SE read data, previously described in Harsimendy *et al*. [9]. Notably, this public data set offers very high coverage (approximately 188×), and SNP calls for a large portion of these loci have also been generated by ABI Sanger sequencing, allowing direct estimation of true positive and false positive error rates. To assess how SNP calling differs by mapping strategy, we computed genotypes using unique (UNI), best-guess (BEST), and total max-*d *(ALL) read mapping strategies (Figure 1b). We also compared our method to Maq [4] and SOAPsnp [3], two alternative methods that generate SNP calls using either unique or best-guess strategies (Text S3 in Additional file 1). Sniper shows excellent, but comparable, performance across the majority of conditions tested, yielding a true positive rate (TPR) of 97.2% and a false discovery rate (FDR) of 0.0%, averaged across the four genotyped individuals and the nine mismatch and read map combinations tested (Additional file 8). However, we found that this region is very atypical of the human genome in general and in fact was especially chosen because of its lack of redundant sequence [9]. Comparison of read maps indicated minimal difference in the number of alignments within the 261-kb template region: averaging over *k *= 1 to 3, the ALL map includes only 0.87% more reads than BEST and 8.25% more reads than UNI (Additional file 9). Moreover, 78% of loci at *k *= 1 show at least 100-fold enrichment of singly to multiply mapped reads, while 87% show a 10-fold enrichment (Figure S4A in Additional file 7). Thus ALL, UNI, and BEST maps are essentially identical for the 261-kb template region, explaining similar performance across methods (Additional file 10). Therefore, we carried out an extensive suite of simulation tests to assess performance on genomes containing multiple paralogous loci (resulting in lower sequence complexity).

We generated a panel of five homozygous diploid synthetic DNA templates that exhibit varying levels of sequence degeneracy (Table 2). These templates were derived from an intrinsically redundant concatenation of 85 ribosomal protein sequences taken from the yeast genome; in fact, these loci are generally difficult to PCR amplify because of this redundancy [18]. We evaluated the performance of Sniper using ALL, UNI, and both BEST guess and BEST no-guess maps on unknown genomic configurations derived from each of these five templates, following the addition of 0.1% polymorphic variation (see Materials and methods). Briefly, we first generated a positive control template (ribosomal protein loci (RPL)) with 85% degeneracy and an average number of alignments per read of 1.88; however, less than 3% of reads are expected to have more than two alignments (Table 2). A negative control (2 × RPL + 0%) consists of two identical copies of RPL, so in principle no SNPs should be identifiable by unique or best read mapping. Finally, three test templates were generated where 2%, 5%, or 10% sequence divergence was added to the second copy of RPL. These synthetic DNA templates represent genomes with two-fold paralogous degeneracy but with decreasing similarity of the paralogs. They have more than two alignments per read on average, 98.6%, 97.9%, and 86.8% degeneracy, and 29.9%, 6.8%, and 1.9% of reads with at least three valid alignments, respectively.

**Table 2:** Data sets used in this study

Region name	Type^a^	Loci^b^	Difficulty^c^	% deg.^d^	APR^e^	% ≥ 3	% ≥ 5	% ≥ 10
Human 261 k	Exp.	261,475	Low	90.4	1.91	0.21	0.05	0.02
Yeast RPL	Sim.	94,678	Low	85.1	1.88	2.60	0.00	0.00
Yeast 2 × RPL +0%	Sim.	189,356	Extreme	97.1	3.01	96.90	3.20	0.00
Yeast 2 × RPL +2%	Sim.	189,356	High	98.6	2.18	29.90	0.52	0.00
Yeast 2 × RPL +5%	Sim.	189,356	Medium/high	97.9	2.05	6.83	0.06	0.00
Yeast 2 × RPL +10%	Sim.	189,356	Medium	86.8	2.01	1.86	0.57	0.00

Genomic templates used in this study: Human 261 k [9]; synthetic concatenation of yeast ribosomal proteins (RPL); perfect tandem duplication of RPL (2 × RPL +0%); tandem duplication of RPL adding 2% sequence divergence to the second copy (2 × RPL +2%); tandem duplication of RPL adding 5% sequence divergence to the second copy (2 × RPL +5%); tandem duplication of RPL adding 10% sequence divergence to the second copy (2 × RPL +10%). See main text for more details. ^a^Whether the sequenced reads were obtained experimentally (Exp.) or by simulation (Sim.). ^b^Indicates the number of positions genotyped. ^c^Determined in the following manner: 100,000 36-nucleotide PE reads were sampled randomly from the template region with perfect fidelity. Sampled reads were aligned to the template with *k *= 3 mismatches. ^d^Percentage of sampled reads with more than one alignment (percentage degeneracy). ^e^Average number of alignments for each sampled read across all reads (alignments per read). The last three columns report the percentage of reads aligning to at least three, five, or ten different loci in the template.

**Figure 3:**
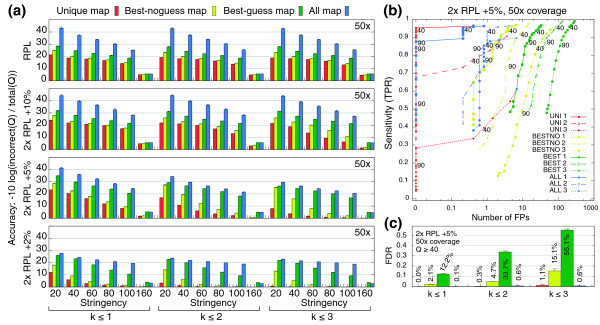
**Genotyping performance on simulated data sets**. **(a) **Bar charts report Sniper genotyping accuracy based on resequencing of four different synthetic genomic DNA templates. Sample genomes were generated from each known template by introducing single nucleotide sequence variation randomly to 0.1%. We simulated 36-nucleotide PE reads from each unknown genome to 50-fold coverage and mapped them to the respective known genomic template according to a unique (red), best no-guess (yellow), best guess (green), or total (blue) mapping strategy using *k *= 1, 2, or 3 mismatches, as shown. Accuracy for each bar was determined using only those genotype loci identified at or above the specified stringency level Q = -10 log_10_(1 - *P*), where *P *is the posterior probability of the MAP genotype at a single nucleotide locus. Error bars represent ± standard deviation across five replicate simulations of generating a new sample genome and simulating reads from this genome. **(b) **Receiver operating characteristic (ROC) styled plot relating genotyping sensitivity, TPR = (TP + TPFG)/(TP + TPFG + FN) (where TP = true positive loci, FN = false negative loci, and TPFG = true position, false genotype loci, to number of false positives (FPs) for simulation results genotyping the moderate difficulty Yeast 2 × RPL + 5% template at 50-fold coverage; as in (a), each point represents the average over five replicates. Points representing stringency levels Q ≥ 40 and Q ≥ 90 are labeled for clarity. **(c) **Bar chart reporting the estimated false discovery rate FDR = (FP + TPFG)/(TP + FP + TPFG) for genotyping the Yeast 2 × RPL + 5% template at 50-fold coverage using Q ≥ 40 confidence. Error bars represent ± standard deviation over five replicates, as in (a). See Additional file 14 for a complete description of estimates.

Results for the positive control and test templates genotyped at 50-fold coverage are shown in Figure 3. (Negative control results are shown in Additional file 11.) In Figure 3a, we report accuracy - (TP + TPFG + TN)/(TP + TPFG + FP + TN + FN), where TP = true positive loci, TN = true negative loci, FP = false positive loci, FN = false negative loci, and TPFG = true position, false genotype loci - as a single performance measure that summarizes all hypotheses as a function of stringency (the minimum posterior probability for a SNP). The total map (ALL) yields maximum accuracy for all 12 comparisons of template and *k *(Figure 3a), all of which show significantly greater accuracy than unique, best-guess or best-no-guess maps (*P *< 0.05). This indicates that the use of multiply mapped reads improves accuracy regardless of template sequence complexity or the number of mismatches used to generate the read map. ALL also shows maximal accuracy over a broad stringency range. The only exception is for the six comparisons at highest stringency (Q ≥ 160) for 2 × RPL +2% and 2 × RPL +5% data sets; however, this occurs because ALL uses a max-*d *strategy that prohibits a few reads that BEST does use. Similar results are found comparing the three read maps at different coverage levels (Additional file 12) with a few notable exceptions: as expected, all of the read maps tend to display similar accuracy for predominantly unique templates at very low coverage (for example, RPL at four-fold coverage); and best-guess maps compare well to total maps for more degenerate templates at high coverage (for example, 2 × RPL +2% at 200-fold coverage), due to greater overall sensitivity and greedy mapping strategy. It is also worthwhile to note that for these simulations, restricting successful alignments to *k *= 1 yields little, if any, decrease in accuracy. This likely results from our use of a relatively low base-call sequencing error rate (0.001) and low levels of polymorphic divergence between sample and template sequence (0.001), such that the expected number of mismatches between a sequenced read and the reference template is less than 1 (see Discussion). That said, these parameter values reflect those determined from the experimental 261-kb human data set.

While achieving the greatest overall accuracy, ALL maintains high sensitivity and specificity, regardless of mismatches or template degeneracy (Figure 3b; Additional files 13 and 14). In contrast, BEST, which shows the most comparable accuracy, yields highest overall sensitivity but also the worst specificity. Computing FDRs for the 2 × RPL + 5% template reveals that ALL performs most optimally on degenerate sequence, with FDRs of 0.1%, 0.6%, and 0.6% with 1, 2, or 3 mismatches, respectively. Despite fully utilizing multiply mapped reads, ALL achieves specificity comparable to UNI for *k *= 1 (0.0%) and *k *= 2 (0.3%) and exceeds UNI at *k *= 3 (1.1%). In contrast, BEST and best-no-guess (BESTNO) maps perform significantly worse (Figure 3c; Additional file 14). We also evaluated performance with BESTNO maps aligned using read quality scores, which revealed slightly improved, but still high, FDRs (Additional file 14). By pooling estimated genotypes across all synthetic templates, mismatches, and coverage levels, we estimated overall error rates for Sniper using each read map strategy at a stringency cutoff of Q ≥ 40 (Table 3). ALL provides a high sensitivity of 0.744 (compared to UNI with 0.648) while maintaining the minimum FDR of 0.027 (compared to UNI with 0.028); in contrast, BESTNO and BEST yield FDRs of 0.176 and 0.400, respectively. Estimates based on a typical experimental design (Q ≥ 40 and 50-fold coverage) and broken down by template reveal even better performance; ALL has an estimated FDR of only 0.58% for *k *= 2 on our most difficult template (compared to 9.57% with UNI; Additional file 14). Furthermore, these estimates are robust to approximately ten-fold discrepancies in sequencing error rate (Text S4 in Additional file 1; Additional file 15). This demonstrates that using multiply mapped reads results in significant improvements in accuracy and specificity compared to single-alignment mapping strategies, without substantial loss of sensitivity compared to greedy approaches. Moreover, read maps containing both unique and multiply mapped reads can, in principle, infer genotypes for all resequenced loci, which is otherwise impossible for all but the simplest genomes.

**Table 3:** Overall performance estimates based on synthetic template simulations

	TPR	FPR	FDR
UNI	0.648	2.475e-5	0.028
BESTNO	0.751	2.213e-4	0.176
BESTNO-Q	0.673	1.939e-5	0.044
BEST	0.816	1.106e-3	0.400
ALL	0.744	3.679e-5	0.027

Sniper performance estimates reflect averages taken across a range of mismatches (1 ≤ *k *≤ 3) and coverage levels (10×, 25×, 32×, 50×, 75×, 100×, 150×) over five independently sampled replicates and four synthetic reference templates (Table 2) at our recommended stringency cutoff of Q ≥ 40. Note, these performance estimates are conservative because the majority of genotyped loci come from non-unique templates. See Additional file 14 for estimates broken down by template. See Figure 1b for complete descriptions of mapping strategies. See Materials and methods for corresponding equations. ALL, unique and multiply mapped reads; BEST, best guess; BESTNO, best no-guess mapping; BESTNO-Q, same as BESTNO but uses read quality values for mapping; FDR, false discovery rate; FPR, false positive rate; TPR, true positive rate; UNI, strictly unique mapping.

### Discovery of novel SNPs in repetitive regions of the human genome

**Figure 4:**
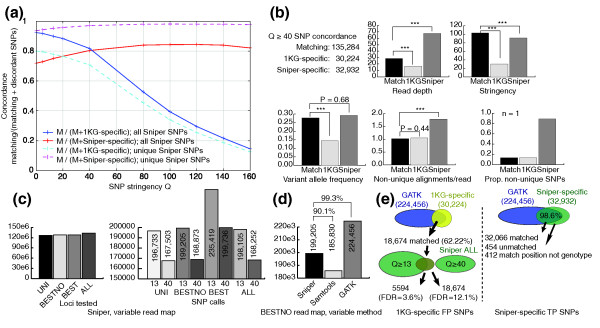
**SNP calls for a high-coverage 1000 Genomes Project individual**. **(a) **Concordance rate (Matching/(Matching + Discordant) SNP calls) across a range of stringency thresholds (MAPthrough *P *< 10^-160^) between Sniper SNP calls and SNP calls from the March 2010 release of the 1000 Genomes Project Consortium for human individual NA19240 chromosomes 20 to 22. Solid lines depict concordance rates using all SNP calls; dashed lines depict concordance rates using the subset of Sniper SNPs covered exclusively by unique alignments **(b) **Estimates of read depth, stringency, variant allele frequency, alignments per read, and proportion of non-unique SNPs averaged over the concordant, Sniper-specific, and 1000 Genomes Project-specific (1 KG) SNP calls (using Q ≥ 40 cutoff for Sniper). Significant comparisons resulting from a two-sample unequal variance *t*-test are indicated. ***P < 10^-10^. No test was performed for the lower right panel due to sample sizes of 1. **(c) **Comparison of Sniper SNPs across mapping strategies, showing total loci assayed and SNP calls at Q ≥ 13 and Q ≥ 40 stringency cutoffs. See Figure 1b for complete descriptions of mapping strategies. **(d) **Comparison of SNPs across SNP calling algorithms (Sniper, SAMtools/Maq, GATK) given an identical read map generated with Bowtie using the best no-guess (BESTNO) strategy. SNPs were filtered using Q ≥ 13 (Sniper and SAMtools) and recommended settings for GATK (DP > 100 || MQ0 > 40 || SB > -0.1). **(e) **Isolation of high confidence false positive (FP; left) and true positive (TP; right) SNPs by intersecting 1 KG-specific and Sniper-specific SNPs with calls from GATK to control for read map differences between this study and the 1 KG study [1]. ALL, unique and multiply mapped reads; BEST, best guess; BESTNO, best no-guess mapping; FDR, false discovery rate; FP, false positive; MAP, maximum *a posteriori*; TP, true positive; UNI, strictly unique mapping.

As a preliminary demonstration of the application of Sniper to experimental data, we identified SNPs in one human individual (NA19240 from Yoruba) using high coverage (approximately 42-fold) SE and PE Illumina sequencing data generated by the 1000 Genomes Project Consortium and compared the results to the final release of pilot project SNP calls (March 2010) [1]. We focused this preliminary analysis on chromosomes 20 to 22 only. Compared to the 165,544 pilot project SNP calls (1 KG SNPs) over these three chromosomes, Sniper identified 168,252 SNPs at moderate stringency (Q ≥ 40) and 198,105 SNPs at lower stringency (Q ≥ 13, *P *< 0.05). Figure 4a shows the proportion of SNPs that agree between Sniper and 1 KG (concordance rate) across a range of stringency thresholds for the Sniper SNPs. As expected, concordance normalized to 1 KG SNPs decreases with stringency, while concordance normalized to Sniper SNPs increases with stringency. The maximum 1 KG-normalized concordance of 92.5% is reached at Sniper's lowest stringency (Q ≥ MAP; simply, maximum *a posteriori *SNP calls); that is, 7.5% (12,416 SNPs) of the 1000 Genomes Project SNPs are not shared with Sniper. Sniper-normalized concordance, on the other hand, is lowest (71.9%) at MAP stringency and increases to a maximum of 84.4% at Q ≥ 120. Sniper's relatively stable curve and high average concordance indicate that a large majority of Sniper SNP calls are high confidence and suggest that most SNPs are most likely to be real. At the same time, many of Sniper's SNP calls (32,932 SNPs at Q ≥ 40) are not concordant with 1 KG. Notably, this discordant portion represents 19.6% of the total set of SNPs identified as significant by Sniper.

Since one key aspect of Sniper's methodology is the use of multiply mapped reads, it is possible that many SNPs harbored in redundant genomic contexts have gone undetected by the 1 KG methodology; in particular, variant calls over approximately 20% of the reference genome were masked primarily due to non-unique read mapping (non-'accessibility') [1]. To evaluate this hypothesis, we first isolated the subset of Sniper calls that only overlapped unique reads and compared genotypes to 1 KG SNPs (Figure 4a). In contrast to all Sniper SNPs, unique Sniper SNPs show greatly increased maximum and minimum concordances of 98.1% (Q ≥ 80) and 93.6% (Q = MAP), respectively. This indicates that when restricted to a comparable read map, Sniper largely recapitulates the same SNPs produced by the 1 KG approach for the unique genomic regions. Consequently, most of the remaining SNPs are non-unique and are not presented in the 1 KG SNP set. However, the maximum 1 KG-normalized concordance compared to these unique Sniper SNPs drops to 80.0% (compared to 92.5%), suggesting either that Sniper is less sensitive than the 1 KG approach or that the 1 KG approach is both less sensitive (since it fails to detect non-unique SNPs) and less specific (because it calls many additional SNPs) than Sniper. To distinguish these possibilities, we characterized the average read depth, confidence, variant allele frequency, degeneracy, and proportion of non-unique SNPs for those non-concordant SNPs exclusively reported by Sniper (32,932 SNPs at Q ≥ 40) or 1 KG (30,224 SNPs). We compared these values to corresponding estimates from the set of 135,284 SNPs concordant between methods (at Q ≥ 40) - assuming that concordant SNPs represent true positive calls - and found a clear disparity between the Sniper-specific and 1 KG-specific sets (Figure 4b). On average, 1 KG-specific SNPs exhibit significantly lower read depth (16.4 alignments/SNP, *P *< 10^-10^), stringency (Q = 30.2, *P *< 10^-10^), and variant allele frequency (14.4%, *P *< 10^-10^) compared to concordant SNPs, suggesting that Sniper did not identify these loci as significant because of weak support for variant alleles. Conversely, Sniper-specific SNPs have significantly elevated average read depth (67.7 alignments/SNP, *P *< 10^-10^) and comparable variant allele frequency (29.3%, *P *= 0.68), while maintaining very high confidence (Q = 91.4). These results suggest that Sniper-specific SNPs have much higher quality than 1 KG-specific SNPs in general. Yet despite these characteristics, Sniper-specific SNPs are 1.7-fold more degenerate than either concordant or 1 KG-specific SNPs (*P *< 10^-10^; Figure 4b); reads align to 1.79 loci on average compared to 1.05 and 1.02 loci, respectively. Using a relaxed criterion for SNP uniqueness (> 25% of overlapping reads must be uniquely mapped), 89% of Sniper-specific SNPs are not unique compared to 13% for concordant and 1 KG-specific SNPs. Thus, the approximately 33,000 SNPs exclusively identified by Sniper exhibit characteristics associated with the putatively true, concordant SNPs, yet notably are located in moderately redundant genomic contexts. At the same time, characteristics of the approximately 30,000 1 KG-specific SNPs suggest that the majority might be spurious, yielding a possible FDR up to 20% for this individual. Notably, this estimate closely matches the approximately 20% error rate expected from subsampling simulations reported by Harsimendy *et al*. for 42-fold coverage (Figure 5b in [9]).

We validated this discrepancy between Sniper and 1 KG SNP calls by comparison with SNPs generated using UNI, BEST, or BESTNO maps. Since BEST and BESTNO maps yield the highest FDRs on our simulated data sets (Table 3), the 32,932 Sniper-specific SNPs (which were generated from the ALL map) are more likely to be valid if ALL yields fewer SNP calls than BEST or BESTNO on this experimental data set. As expected, the total number of loci with positive read depth varied by mapping strategy, with ALL yielding the most hypotheses and UNI the least (Figure 4c). In contrast, BEST reported the most significant SNPs while the other maps reported comparable numbers; this is true at both moderate (Q ≥ 40) and lower stringency (Q ≥ 13; Figure 4c). Thus, ALL does not predict more SNPs compared to maps with elevated FDR, further suggesting the validity of Sniper-specific SNPs. An alternative possibility is that Q ≥ 40 is an overly stringent cutoff, thus predisposing Sniper to miss true SNPs (false negatives). In support of this, decreasing stringency increases concordance between ALL and 1 KG calls (Figure 4a). To control for this putative loss of sensitivity, we compared 1 KG-specific SNPs to Sniper SNPs from the more sensitive BEST and BESTNO maps. However, at Q ≥ 40 only 1.54% of 1 KG-specific SNPs match BEST SNPs (*n *= 465), and only 0.82% match BESTNO SNPs (*n *= 248). While reducing stringency to Q ≥ 13 does increase concordance substantially to 45.2% (BEST) and 44.9% (BESTNO), these are nevertheless comparable to concordance with ALL at Q ≥ 13 (44.7%, *n *= 13,491). Thus, even assuming that all SNPs matching at this low stringency are true, the argument of decreased sensitivity fails to explain the majority of 1 KG-specific SNPs.

Since our Bowtie read maps likely differ from the map used to generate the 1 KG SNPs (for example, we did not control for biased SNP calling near possible indels), we also assessed the effect of using different read maps to explain the discrepant SNP calls. We compared Sniper calls with those produced by GATK [19] and Maq/SAMtools [4,20] (using the authors' recommended filtering criteria) given the same BESTNO map as input. Both methods largely agree with Sniper (Q ≥ 13) calls (90.1% and 99.3% concordance, respectively; Figure 4d), indicating that the discrepancy between Sniper and 1 KG is not due to implementation-specific factors independent of the read map (for example, filtering criteria). While at best Sniper matches 44.9% of 1 KG-specific SNPs (see above), GATK matches 62.2%, indicating that after controlling for read map differences, GATK (notably one of the methods used to generate the 1 KG SNPs) yields results that are much more similar to 1 KG than does Sniper. Of course, since GATK also shows partial concordance with 1 KG SNPs, this does indicate there is a read map effect (that is, our Bowtie maps differ from the 1 KG map). We controlled for this difference in two ways. First, we checked for potential indels using GATK and generated a new read map based on local realignment of reads near indels. However, none of GATK's SNP calls were altered by this procedure. Second, we restricted attention to the subset of 18,674 1 KG-specific SNPs that were matched by GATK (Figure 4e); aside from minimizing the read map effect, this provides a further control for indel bias because these SNPs were thus identified with a global-alignment map. Only 30.0% of this reduced set of 1KG SNPs (5,594 at Q ≥ 13) is concordant with Sniper, while increasing stringency to Q ≥ 40 results in 0% concordance. Thus, after controlling for read map differences, indel bias, and potential false negatives 13,000 to 18,000 of the 1 KG SNP calls are discordant with Sniper. Given this result, we estimate that the FDR for 1000 Genomes Project SNPs lies between 3.63% and 12.13% when restricted to the uniquely accessible portion of the human genome. Notably, this FDR range closely matches that provided by Harsimendy *et al*. [9] based on first-generation sequencing technology and older SNP calling methodology, indicating little if any improvement in the rate of false SNP discovery has been made using newer sequencing platforms or improved methodology. Furthermore, the 1000 Genomes Project Consortium itself reported that the FDR for their low-coverage SNPs (determined by consensus of three different methods) was reduced by 30 to 50% compared to any single method, while high-coverage 1 KG SNPs (that is, 42-fold coverage) were only based on the consensus of two methods. They also used variants in the dbSNP database to estimate a FDR around 5% for SNP calls from high-coverage individuals [1]. Thus, we conclude that current, sophisticated methods that rely on singly mapped reads for variant discovery still face a significant FDR, even when carefully masking redundant genomic contexts. In contrast, Sniper yields an estimated FDR of only 0.27% at 42-fold coverage, averaged across both unique and redundant contexts and using a similar two-mismatch cutoff (Additional file 14). Thus, by integrating unique and non-unique reads, Sniper appears to provide significantly greater specificity than the 1 KG methodology, even using a nominal significance cutoff.

Next, we assessed whether the 32,932 Sniper-specific SNPs not present in the 1 KG data set also exhibit a read map effect. We compared them to SNP calls generated by GATK (using the BESTNO map) and by Sniper (using the BEST map) and found that 98.6% and 98.3% are matched, respectively. Thus, the vast majority of Sniper-specific SNPs can be explained by a difference in read maps, notably through the use of redundant positions masked in the 1 KG results. Regardless, concordance of Sniper-specific SNPs by GATK validates the majority of the Sniper-specific calls. Moreover, 454 SNPs were not identified by GATK, suggesting a possible increase in SNP occurrence rate by 0.2% (Figure 4e); 412 other variants are identified by GATK, but at these positions it proposes differing genotypes. Similar to the bulk of Sniper-specific SNPs, these discordant loci average 1.41 non-unique alignments per read, confidence of 80.7, depth of 417 reads (median 62), and variant allele frequency of 0.245. In contrast, analyzing these loci using the BESTNO map shows reduced confidence of 60.6, reduced depth of 121 reads (median 25), and reduced variant frequency of 0.130, suggesting that the Sniper-specific genotypes are correct. Thus, for SNPs covered by a mixture of unique and non-unique reads, accurate genotyping requires utilizing the information from all alignable reads.

We then queried the HapMap SNP collection [21] to determine whether any of Sniper's SNP calls could be independently validated. Overall, HapMap provides 82,381 SNP loci for individual NA19240, 68,237 of which (82.8%) are present in the set of 168,252 Sniper (Q ≥ 40) SNPs (and 90.2% using Q ≥ 13). Another 837 variants match in position but differ in genotype, and 1,064 variants are genotyped by Sniper but lack a genotype in the HapMap collection. For comparison, GATK matches a similar, but slightly lower, number of HapMap SNPs (68,179) and predicts discordant genotypes at 853 additional loci. Notably, GATK matches 820 of Sniper's 837 discordant SNPs (98.0%), suggesting that corresponding HapMap alleles may be incorrect. Thus, compared to HapMap SNPs, Sniper using the ALL map shows slightly greater sensitivity than GATK using a BESTNO map. Focusing on the subset of 32,932 Sniper-specific SNPs not found in the 1 KG data set, 1,577 positions (4.8%) have a matching genotype, and 227 variant positions differ in genotype; in contrast, none of the 1 KG-specific SNP calls are present in HapMap. Finally, only two of the 454 SNPs predicted only by Sniper were found among HapMap variants, one of which showed a different genotype (Additional file 16). Of the 412 SNPs with discrepant genotypes compared to GATK, 28 were also identified, half of which disagree with the HapMap genotype; note that these 14 SNPs have nearly twice as much confidence and significantly lower degeneracy than the 14 SNPs that do match in HapMap (*P *= 10^-4^), suggesting they are valid (Additional file 16). Thus, since Sniper and GATK show similar concordance with the HapMap collection over the three chromosomes we analyzed, and since Sniper-specific SNPs show much better concordance compared to 1 KG-specific SNPs, we conclude that the majority of Sniper SNP calls are likely to be correct. Also, since chromosomes 20 to 22 represent only approximately 3.5% of the entire human genome, we anticipate that Sniper would identify approximately 13,500 SNPs in redundant genomic contexts throughout the genome; notably, this estimate only considers SNPs that would be unidentifiable by methods utilizing unique reads alone. Moreover, given the sensitivity estimates described in our study, we anticipate that human genome data sets incorporating greater than 42-fold sequencing coverage would identify an abundance of additional SNPs in both unique and redundant genomic contexts.

## Discussion

The primary aim of genome resequencing studies is to discover accurately all loci that are polymorphic in a population. To this end the methods used to discover polymorphisms using NGS data aim to consider the possibility of variation at any and all genomic loci and to accurately report genotypes for all variant loci. Existing methods rely on singly mapped near-optimal alignments to achieve high specificity, whether by a strict definition of uniqueness or by approximation (Figure 1b). Consequently, NGS studies typically under-utilize available sequencing data, especially reads that overlap the true, unknown polymorphisms - precisely the loci under investigation. While exclusive use of unique reads does generally yield fewer false calls over unique contexts, this strategy presents two primary drawbacks: SNP loci often occur in mildly redundant contexts, resulting in biased genotyping estimates at true SNP loci and increased rate of false SNP discovery (Figure 4e); and significant portions of a reference genome simply may not be mappable, or are only mappable at a low coverage rate, due to inherently redundant, paralogous sequences. Thus, redundant genomic contexts are a major factor limiting both sensitivity and specificity of SNP discovery. While adopting a more lenient unique mapping strategy (such as best guess or best no-guess) does increase the number of mapped loci compared to a strictly unique strategy, this risks introducing sampling bias among repetitive elements and paralogs and, given sufficiently short reads or high sequencing error rates, can yield spurious alignments. Thus, lenient mapping strategies tend to improve sensitivity in unique genomic contexts at the cost of an elevated FDR. To reduce false calls, current approaches have supplemented careful read mapping and genotyping with quality score recalibration, cumbersome multifaceted filtering strategies, and masking of redundant loci. Alternatively, as we have shown using our novel method Sniper, simply considering multiply mapped reads and unique reads using probabilistic integration can successfully provide both maximal coverage, accuracy, and specificity at the cost of slightly reduced sensitivity. Given available sequencing data, our method evaluates both unique and redundant loci in a unified, Bayesian probabilistic framework and successfully refrains from calling false SNPs by correcting for sequence degeneracy across paralogous loci. Our model framework offers a complete representation of the problem of SNP discovery using NGS data, allowing for further principled generalizations. Here, we limited our implementation to the most critical parameters only. However, our model's generality allows for much greater specification to incorporate, for example, platform-specific error models and more realistic models of population heterozygosity, not to mention inclusion of quality score recalibration and other sophisticated data corrections.

In this study we focused our analysis on a variety of quality-blind mapping strategies. Incorporating base call qualities into single-alignment maps can help (Additional file 14), but our analysis of existing 1000 Genomes Project data strongly argues that it is still prone to generating false positives at a rate comparable to older methods applied to older sequencing chemistry (FDR ≈ 3 to 12%). In our current implementation, base-call qualities were incorporated into SNP probabilities but not into prior template location probabilities. Yet, we found relatively optimal performance using Sniper over a broad range of conditions. Without the Sniper strategy, at least 15% of the human genome would not be assayable using a single-alignment read map without risking unwieldy increases in FDR (Figure 3c; Additional file 14).

Since genome resequencing remains a costly exercise, most studies are currently limited to low or moderate sequence coverage. Thus, optimal use of available data is crucial. Using our method, we find that between 10-fold and 25-fold coverage is required to be able to identify a simple majority of true SNPs in realistic data sets, while at least 50-fold coverage is desired to approach full sensitivity with confidence (Additional files 10 and 13). Since reads that align to multiple locations are informative for all overlapping loci, our method tends to boost accuracy at low coverage (≤25-fold) without sacrificing specificity (Additional files 12 and 13). Further gains in accuracy may be achieved by increasing the maximum number of multiple alignments *d *+ 1 and by increasing the number of allowed mismatches per locus *k*, both of which will increase the number of mapped reads and the number of alignments per read. It is important to note, however, that increasing either *k *or *d *can end up reducing variant sensitivity *per se*, at the expense of accuracy. As *k *and *d *increase, information from a single read becomes diluted across multiple alignments. For example, more NGS reads will typically map uniquely to a given template using *k *= 1 than with *k *= 2. As discussed in our results, variant sensitivity is maximized when using a value of *k *that maximizes the genome-wide ratio of average read depth versus average number of alignments per read. Of course for a given *k*, one can approximate this simply by reducing the number of alignments returned by the mapping algorithm (that is, by reducing the parameter *d*), thereby concentrating sequence information over fewer loci; in the limit where *d *= 0, this becomes the best-guess strategy. As we have shown, this approximation does work well for long read lengths over unique genomic sequence. However, unless non-unique regions of the genome are explicitly masked, this strategy will fail on realistic data sets. While recent methods have been developed to improve specificity further by integrating unique mapping with imputation using population-based SNP information [22] - essentially how low-coverage 1000 Genomes Project data have been analyzed [1] - this strategy still cannot be used to investigate redundant or paralogous loci.

Finally, our analysis of synthetic genomic templates (Additional files 12 and 13) may be used to guide selection of an appropriate stringency level for novel genome resequencing studies. While our method is highly accurate in principle because the probability of a SNP at any given nucleotide site is estimated within the context of that site's paralogous loci, many factors influence genotyping performance, including coverage level, unknown sequencing errors, number of mismatches, reads missing due to library preparation bias, the short-range correlation between adjacent sites due to shared overlapping reads, and the long-range correlation due to positional paralogy. Our results suggest that, while maximum sensitivity is achieved at or below a baseline stringency of Q ≥ 13 (*P *< 0.05), maximum specificity is achieved at Q ≥ 90 (*P *< 10^-9^), especially for low complexity sequences. We find that a good balance is generally achieved using a Q ≥ 40 cutoff (*P *< 10^-4^), which is approximately the stringency needed to correct a *P *< 0.01 model error for the correlation across adjacent loci using a Bonferroni-style multiple test correction. To obtain a more comprehensive cutoff, our implementation of Sniper also applies a broader Bonferroni-style correction across both adjacent and paralogous sites by determining the necessary stringency level for each individual SNP based on that site's overall read degeneracy.

## Conclusions

In this paper, we presented a new probabilistic method for SNP discovery using NGS data. But regardless of the SNP inference method itself, we found several rules-of-thumb applicable to any NGS-based study: (1) use all mappable reads to increase accuracy; (2) use long, PE reads with short template lengths to minimize multiply mapped reads; (3) generate at least 25-fold coverage, ideally ≥50-fold coverage to minimize sampling error; (4) use a posterior probability error threshold of *P *< 10^-4 ^to maintain high SNP confidence; (5) set *k *≤ 0.03 mismatches per base of each read for sequence alignment. Note that *k *is most likely platform-dependent. Local fluctuations in sequencing quality, non-constant sequencing error rates across the length of an individual read, use of a reference genome containing sequence divergence in excess of 0.1%, and occurrence of multiple SNPs within one read length may necessitate larger *k*. So, in general, one should set *k *≈ 1/*|r| *+ *e_max _*+ π, where *|r| *is the read length, *e_max _*is the maximum expected sequencing error rate per base per read, and p is the expected divergence per site between reference and (unknown) sample genomes.

Genome resequencing using multiply mapped reads improves our ability to discover novel SNPs precisely without loss of sensitivity. Our method is especially useful for polymorphism detection in redundant genomic contexts, such as repetitive elements, paralogous genes, segmental duplications, and copy number variants, which are typically ignored because of their tendency to generate multiply mapped reads. The probability model also contains explicit components for sequencing errors and library template biases that may be parameterized in more detail in the future, allowing the method to be extensible and adaptable for specific sequencing platforms.

## Materials and methods

### Read sampling strategy

For perfect resequencing (Figure 2a), 16 sequencing experiments were simulated by generating independent sets of 25 × 10^6 ^PE reads without addition of base-call errors or sequence divergence; read sets were simulated for four read lengths (30, 60, 90, and 120 nucleotides) and four template, or fragment lengths (means of 250, 500, 750, and 1,000 nucleotides), yielding 0.47, 0.94, 1.41, and 1.88-fold coverage genome-wide, respectively. Fragment lengths were sampled for each PE read using a Gaussian distribution with mean *μ *and standard deviation 30, where *μ *is 250, 500, 750, or 1,000. Read quality values were sampled for each nucleotide along a read according to an empirical distribution (see next section, 'NGS data sets'). Reads were mapped allowing up to 1, 2, 3, or 3 mismatches, respectively. PE reads were aligned first, then unmapped reads were aligned individually as SE reads. For error-prone sequencing (Figure 2b), four sequencing experiments were simulated (30-, 60-, 90-, and 120-nucleotide read lengths) by generating independent sets of approximately 180 × 10^6 ^PE reads (approximately 4.7-fold coverage) followed by random addition of 1% base-call errors. Fragment lengths and quality values were determined as described above. All reads were mapped using *k *= 2 mismatches.

### NGS data sets

Analyses were performed using both experimental and simulated NGS data. We obtained 36-nucleotide SE Illumina 1G reads sequenced from 261 kb of genomic template for four *Homo sapiens *individuals (NA17156, NA17275, NA17460, NA17773) from Harsimendy *et al*. [9]. Simulated 36-nucleotide PE reads were generated from known genomic sequences, following the addition of sequence variation to constitute an unknown sample genome (see next section, 'Synthetic genomic DNA templates'). The chromosome and starting position of each PE read were chosen randomly. The insert size for each read was drawn from a Gaussian distribution with mean 250 and standard deviation 30, following standard experimental guidelines for the Illumina platform. Each pair of sequences was copied from the genomic template using the randomly sampled coordinates, reading from each terminal boundary inward. Sequencing errors were introduced randomly at each sequenced position at a rate of 0.001 errors per position. A quality score was associated with each base by drawing a Phred score from a Gaussian distribution with standard deviation 10 and position-specific mean determined using 36-nucleotide PE Illumina data from a *Saccharomyces cerevisiae *genome we generated (data not shown). The following 36 means were used: 32.8, 32.6, 32.5, 32.5, 31.9, 31.8, 31.7, 31.8, 31.5, 31.3, 31.2, 31.0, 30.5, 30.4, 29.9, 30.0, 29.3, 29.5, 29.3, 29.2, 29.1, 29.3, 29.4, 29.6, 29.3, 29.3, 28.6, 28.4, 28.0, 27.7, 27.2, 26.3, 26.2, 26.0, 24.8, 24.2. The magnitude and pattern of these quality scores generally match those calculated from our human NGS data. Simulated reads were generated using up to one of nine different genome-wide average coverage depths: 4-fold, 10-fold, 25-fold, 32-fold, 50-fold, 75-fold, 100-fold, 150-fold, 200-fold. Five independent replicate read sets were generated for each reference genomic template and for each coverage level.

NGS data from the 1000 Genomes Project were downloaded from [23]. For redundancy structure analysis, we selected three PE data sets for each of twoindividuals (NA12892, CEU; NA19238, YRI) that show differences in read length (36 to 76 nucleotides) and template size (175 to 458 nucleotides) (Additional file 3). Reads were mapped using two mismatches as described above; PE reads were mapped using the specified insert size boundaries (Additional file 3). For full genotyping, we downloaded and mapped 245 'high coverage' PE data sets produced from one individual (NA19240, YRI). All reads were mapped allowing only *k *= 1 mismatch and *d *= 5 alignments per read against the hg18/NCBIv36 reference human genome [24]. Mapping of mixed-length reads was permitted. The final release of SNP calls generated by the 1000 Genomes Project Consortium were used for comparison (March 2010) [25]. SNP calls from the HapMap project were downloaded from [26].

### Synthetic genomic DNA templates

A panel of five synthetic genomic DNA templates were generated (from *S. cerevisiae *S288c genomic sequence) that exhibit varying levels of sequence degeneracy (Table 2). Each synthetic template is considered homozygous diploid. Five of the templates are based on a common 94,678-nucleotide sequence that is the concatenation of 85 RPL from the yeast genome, including 500-nucleotide flanking sequence upstream and downstream of each locus. For the other RPL-derived templates, two copies of the RPL sequence were concatenated (totaling 189,256 nucleotides), and varying amounts of divergence (0%, 2%, 5%, and 10%) were added randomly across the template. Sample genomes were obtained from these synthetic templates by introducing sequence variation (corresponding to population-level genomic divergence) randomly at each position at a rate of 0.0005 heterozygous polymorphisms per locus and 0.0005 homozygous polymorphisms per locus, for a total proportion of variation of 0.001. This yielded 94 SNPs for the RPL template and 188 SNPs for the four different 2 × RPL templates. These derived genomic templates represent the unknown genomic configurations whose sequence we wish to infer.

### Read map generation using sequence alignment

Sequence reads were aligned to a reference genome using Bowtie version 0.11.3 [27] (for Sniper and Maq analysis) or SOAPaligner version 2.20 [3] (for SOAPsnp analysis). NCBI v36 (hg18) of the human genome was used as the known reference genome to align human experimental reads [24]. Synthetic genomic DNA templates were used to align simulated reads. Three different approaches were used to map sequence reads to a reference genome: unique (UNI), best-guess (BEST), and total (ALL). The UNI map consists of reads that align exactly once to the reference genome, given the specified number of mismatches *k *(in Bowtie, '-a -m 1 -best'). The BEST map consists of reads having one or more valid alignments, but only a single alignment (the one with fewest mismatches, '-k 1 --best') is used. The ALL map consists of all reads with one or more valid alignments. In principle, the top *d *+ 1 alignments for each read ('-k *d*+1 --best') or simply all reads ('-a') should be returned for ALL; however, either option results in an explosion in the size of the read map (for the human genome especially), likely due to reads aligning to very low complexity repetitive elements. To avoid this explosion, we instead focused on reads with a maximum of *d *+ 1 alignments ('-a -m *d*+1 --best'), rather than the top *d *+ 1 alignments. These three read mapping approaches are parameterized by *k*, the number of mismatches allowed to map each read to the reference genome, and *d *+ 1, the number of genomic loci to which a read can align. For UNI and BEST *d *+ 1 = 1; for ALL *d *+ 1 = 200 (approximately the expected genome-wide sequencing coverage for our human data) unless otherwise stated. Read maps were generated using *k *= 1, 2, or 3. Sequence quality scores were ignored during the alignment process ('-v *k*' mode for Bowtie). When using SOAPalign, the BEST approach was used exclusively ('-M 4'). Default settings for Maq and SOAPalign were used otherwise. For PE read mapping, valid alignments allow *k *mismatches for each of the paired reads as well as valid spacing between the mapped locations of each mate pair. A range of 150 to 300 base pairs was used to define valid spacings. PE read maps were obtained by aligning PE reads first and then aligning the remaining unmapped reads as if they were SE reads.

### Genotyping overview

Since all unknown sample genomes are diploid, ten genotype values (AA, AT, AC, AG, TT, TC, TG, CC, CG, GG) are considered for every genomic locus (position). For all loci with read depth >0, a genotype call was produced as the genotype having MAP probability. Significant SNPs (that is, those significantly different from the corresponding reference genotype) were determined by removing those loci whose MAP probability did not meet a specified stringency cutoff *Q*.

### Performance comparisons

ABI Sanger data were downloaded from dbSNP (handle: RSG_JCVI) [28] for the four individuals genotyped in Harsimendy *et al*. [9]: NA17156, NA17275, NA17460, NA17773. Chromosomal positions for these SNPs were obtained by nucleotide BLAST against build v.36 (hg18) of the human genome [24]. We utilized two benchmark data sets for performance comparisons, called *Sanger *and *Sanger\NGS*. For the *Sanger *benchmark, the total number of positions sequenced by ABI Sanger and that overlap genomic subsequences selected by Harsimendy *et al*. [9] are 449, 206, 279, and 250 for the four individuals, respectively, totaling 1,184 loci; this includes polymorphic and non-polymorphic positions (and excludes unknown alleles 'N'). From these loci, Sanger sequencing identified 111, 60, 79, and 94 variant positions, totaling 344 SNPs. The *Sanger\NGS *benchmark is composed of the subset of *Sanger *loci where genotype calls are identical across the three NGS platforms (ABI SOLiD, Roche 454, and Illumina Solexa) as reported by Harsimendy *et al*. [9]. This set includes 253 SNPs, 73, 50, 66, and 64 SNPs for each individual. For simulation performance comparisons, SNPs identified from simulated read sets were compared to the true polymorphisms that we introduced randomly to each of the synthetic genomic DNA templates. We used the following performance metrics (where TP = true positive loci, TN = true negative loci, FP = false positive loci, and TPFG = true position, false genotype loci): true positive rate, TP/(TP + FN); false negative rate, FN/(TP + FN); false positive rate, (FP + TPFG)/(FP + TPFG + TN); false discovery rate, (FP + TPFG)/(TP + FP + TPFG); and accuracy, (TP + TN)/(TP + FP + TPFG + TN + FN). Simulated accuracy values are reported on a log scale, using a log transformation similar to phred scores: -10 log_10_(1 - Accuracy). Transformed accuracy values range from 0 to + ∞. For example, an accuracy value of 0.99999 (1 - 1e-5) becomes 50.0 on this scale. Thus, a 10-unit increase in accuracy on this scale (for example, from 20 to 30) represents a 10-fold increase in the original accuracy proportion (from 0.99 to 0.999). Human accuracy values (Additional file 10) are reported as -log_10_(1 - Accuracy) without the coefficient of 10. Thus, an accuracy value of 0.96 corresponds to a transformed accuracy of 1.4.

### Likelihood model

Read likelihoods are modeled as:

where *S^G ^*denotes the locations of the reference genomic substrings *s_j _*whose Hamming distance is at most *k *bases from the read *r_i_*. The probability of read *r_i _*originating from a genomic substring *s *was computed as a weighted average of a global error model computed from binomial probability *P_bin_*(*n,k,e*) and a read-specific quality score probability *P_read_*(*q*) as follows:

For *P_bin_, n *= |*r*| is the sequence read length, *k *= selected number of alignment mismatches, and *e *= expected per-locus sequencing error rate, where *e *= 0.0003 was selected by empirical testing; note this is three-fold smaller than Illumina's advertised error rate (*e *< 0.001). The parameter *e *can be estimated, for example, as the mean or median error-probability using read quality scores; however, this approach proved too stringent for our human data. The parameter *P_read _*was computed using a read's per-base quality scores:(9)

where *p_i _*is the position-specific probability of sequencing error. We selected a weighting factor *λ *= 0.67, which maximized agreement with human Sanger genotype calls. (Again we found Illumina quality scores to be slightly conservative for our data.) In general, the weighting factor *λ *can be used to tune read probabilities between global and read-specific models, although we found that results were very robust to variation in *λ *(data not shown).

### Prior probability model

A weighted prior probability distribution was used, consisting of four possibilities: Pr{homozygous reference} = *P*(*G_xy_*) = 1 - ϑ - ϑ^2^; Pr{heterozygous with 1 alternative allele} *P*(*G_x'y_*) = ϑ/2; Pr{homozygous alternative} = *P*(*G_x'x'_*) = ϑ/2; Pr{heterozygous with 2 alternative alleles} *P*(*G_x'y'_*) = ϑ^2^. ϑ = 0.001 was used for human and simulated data to match expected genome-wide heterozygosity.

### Software implementation and computational complexity

Sniper (SNP Identification using Probability of Every Read) is an implementation of our algorithm written in Python (and tested under versions 2.5 and 2.6); core computations have also been implemented in C for (optional) runtime improvement. Sniper takes as input a set of raw sequence files in fasta or fastq format along with a map file that specifies for each sequence file if it is single or PE, the reference genome to map against, and whether multiple files (lanes) should be pooled together. Sniper can perform all steps of analysis, including read map generation, organization of read maps into singly mapped and multiply mapped partitions, and SNP calling. Although Sniper is designed to use Bowtie for read alignment, any alignment program can be specified, as long as the read map output is stored in a SAM-formatted file [24]. The SNP discovery aspect of the algorithm has a runtime complexity of *O*(*n |G|*), where *|G| *is the length of the reference genome and *n *is the expected per-locus read depth (which is a function of *k *and *d*). Memory complexity is *O*(*|G|+|M|*), where *|M| *is the size of the read map index for non-unique alignments; this is implemented as a dictionary of read IDs and byte locations in the read map relevant to the specified genomic region. In practice, |M| can range from 0 bytes (if stream-loaded from disk) up to a few gigabytes depending on genome and data set. Also, a subset *d*' ≤ *d *of all alignments per read can be loaded (using the -d <INT> flag). To reduce computation time, genotyping can be subdivided into separate processes (for example, by chromosome or subsets thereof). Sniper has built-in support for multiprocessor job distribution on a single machine as well as distributed across machines using the Sun Grid Engine [29].

To calibrate the runtime of Sniper, genotyping a 250e6 nucleotides sequence at approximately 70× coverage using the hybrid Python + C implementation took approximately 85 hours using a single 3.3Ghz 64-bit Intel Xeon CPU. Extrapolating, we anticipate processing the entire human genome at similar coverage would take approximately 50 days using a single CPU. However, this could be reduced to 13 days on a four-CPU machine, or less than 1 day if distributed on a computing cluster. Note that overall sequencing coverage and the values for parameters *k *and *d *will affect runtime significantly. Genotyping with *d *= 2 compared to *d *= 200 reduced runtime by about 50% on the Harsimendy *et al*. data. Our software implementation is available on our web site [30] and as Additional file 17 of this article.

## Abbreviations

FDR: false discovery rate; NGS: next-generation sequencing; PE: paired-end; RPL: ribosomal protein loci; SE: single-end; SNP: single nucleotide polymorphism; TPR: true positive rate.

## Authors' contributions

JK conceived and developed the model; DS implemented the model and performed the experiments and analysis. DS and JK wrote the paper. Both authors have read and approved the final manuscript for publication.

## Supplementary Material

Additional file 1 **Texts S1 to S4**. Text S1: description of the analysis of repetitive elements contributing to non-unique alignments. Text S2: details of our Bayesian probability model for SNP detection. Text S3: performance estimates based on comparison to the Sanger validated data set reported in Harsimendy *et al*. [9]. Text S4: performance estimates obtained when varying the expected base-call sequencing error rate parameter compared to actual error rate. Click here for file

Additional file 2 **Figure S1 - simulated paired-end read multiplicity distributions for the human genome**. The number of valid alignments against the *Homo sapiens *genome is reported as a proportion of the 2 × 10^6 ^randomly sampled PE reads used in alignment, varying fragment length (250, 500, 750, or 1,000 nucleotides) and read length (30, 60, 90, or 120 nucleotides). The proportion of read multiplicity averaged over reads of the same length is shown in each figure. Click here for file

Additional file 3 **Table S1 - redundancy structure analysis for human NGS data**. This Excel file describes the number of reads and number of alignments for each read map type (unique (UNI), best-guess (BEST), and total max-*d *(ALL)) associated with the human genome. Click here for file

Additional file 4 **Figure S2 - examples of false read mapping**. False read mapping occurs when a sequenced read is incorrectly aligned to its reference sequence (that is, to the incorrect location in the genome). This is most likely to occur in the presence of closely related sequences existing in replicate in the reference sequence and can result from either **(a) **SNP occurrence or **(b) **base-call sequencing error in the sample genome, such that the similarity between reads containing a variant (or false) allele and the reference genome decreases at one locus and increases at another (false) locus. Instances of false mapping consequently decrease the chance of a SNP call at a true locus and increase the chance of a false SNP call at the wrong locus. Click here for file

Additional file 5 **Table S2 - complete spurious mapping statistics**. This Excel file contains read map statistics for reads that overlap SNP loci. Click here for file

Additional file 6 **Figure S3 - relationship between posterior probability and read degeneracy**. Box and whisker plots showing the distribution of posterior probabilities Q (stringency) for all SNPs identified in each of five replicates for two different simulations (ribosomal protein loci (RPL) and 2 × RPL + 10%), grouped by per-locus degeneracy. For example, the 0 ≤ <*d *< 0.1 group contains loci with at least a 1/10 ratio of alignments at another locus versus alignments overlapping the locus of interest. Box plots represent the entire distribution of Q values for each degeneracy bin, where the red line indicates median and the box indicates the 25th and 75th percentiles. SNPs were obtained using the 25-fold coverage simulations allowing *k *≤ 1 mismatch. Click here for file

Additional file 7 **Figure S4 - per-locus distributions of degeneracy**. Cumulative distributions of per-locus degeneracy are shown for each of the six reference genomic DNA templates used in this study. Degeneracy is defined as the ratio of *d*, the number of alignments for a read that overlap loci other than the locus of interest, to the read depth at a locus of interest. (Alternatively, Alignments/Reads - 1.) For example, a ratio of 1 indicates that every read overlapping the locus of interest has two valid alignments in the reference genome. Loci are binned into 12 groups and the cumulative frequency of all loci is reported at each degeneracy group. Estimates are shown using the ALL read map with *k *= 1, 2, and 3 mismatches. Click here for file

Additional file 8 **Table S3 - complete statistics for human genotyping performance comparison**. This file contains performance statistics based on the Harsimendy *et al*. [9] human data set for Sniper, Maq, and SOAPsnp at different stringency levels for ALL, UNI, and BEST read map types; total SNP loci identified; and putative novel SNPs identified by Sniper. For each program and read map approach, genotypes for four individuals were compared to those determined by ABI Sanger sequencing. The benchmark set (Sanger \ NGS) contains 253 SNPs. True positive rates (TPRs) and false discovery error rates (FDRs) were estimated from these comparisons (top rows). In parentheses, from left to right: matching genotypes; positions identified as SNP but differing in genotype; SNPs not identified by Sanger; Sanger SNPs not identified by program. Sniper SNPs are reported using a stringency threshold on the phred-like posterior probability: Q = -10 log_10_(1 - *P*), where Q ≥ 13 (*P *< 0.05). Maq SNPs were generated using Q ≥ 13 minimum consensus quality, Q_adj _≥ 13 minimum adjacent quality, and prior probability of SNP *P_SNP _*= 0.001, with default settings otherwise. Soap SNPs were generated using default settings, '-t -u -n', and a 2:1 transition:transversion ratio for prior probability, and *P_SNP _*= 0.001. Four SNPs are predicted by Sniper, Maq, and SOAPsnp for *k *= 2 mismatches, identified by comparison to Sanger benchmark data (Additional file 8). Statistics were generated using Sniper. Click here for file

Additional file 9 **Table S4 - read map statistics for the Harsimendy *et al*. NGS data set**. This Excel file details our read maps for the Harsimendy *et al*. [9] human NGS data. Click here for file

Additional file 10 **Figure S5 - human genotyping performance across coverage levels**. Accuracy bar charts and Receiver operating characteristic (ROC)-style curves for human SNP identification at six coverage levels. Reads from one individual (NA17156) were subsampled randomly from the complete approximately 188-fold coverage data in five replicates. Each subsampled read set was independently aligned to the human genome using ALL, UNI, or BEST maps with *k *= 1, 2, or 3 mismatches and genotyped using Sniper. **(a) **Bar charts reporting genotyping accuracy for each condition. Error bars show ± standard error of the mean. **(b) **ROC-style curves are shown as 1 - accuracy versus sensitivity. Three stringency levels (Q ≥ 13, 40, 90) are shown for each curve. Click here for file

Additional file 11 **Figure S6 - simulation negative control. (a) **Accuracy bar charts and **(b) **ROC-style curves showing performance of ALL, UNI, and BEST maps on our negative control synthetic DNA template (2 × RPL +0%). Five unknown sample genomes were generated from each reference template by adding SNPs randomly to a proportion of 0.001. Read sets were sampled from each sample genome to one of four coverage levels (25-fold, 50-fold, 100-fold, 200-fold). Read sets were independently aligned to their respective reference genome using ALL, UNI, or BEST maps with *k *= 1, 2, or 3 mismatches and genotyped using Sniper. **(a) **Bar charts report genotyping accuracy for each condition. Error bars show ± standard error of the mean over five replicates. **(b) **ROC-style curves are shown as the number of false positive calls versus sensitivity. Three stringency levels for Q (13, 40, 90) are shown for each curve. Click here for file

Additional file 12 **Figure S7 - genotyping accuracy for simulated data**. Bar charts are shown reporting SNP identification accuracy on four synthetic genomic DNA templates (RPL, 2 × RPL +2%, 2 × RPL +5%, 2 × RPL +10%). Five unknown sample genomes were generated from each reference template by adding SNPs randomly to a proportion of 0.001. Read sets were sampled from each sample genome to one of nine coverage levels (4-fold, 10-fold, 25-fold, 32-fold, 50-fold, 75-fold, 100-fold, 150-fold, 200-fold). Read sets were independently aligned to their respective reference genome using ALL, UNI, or BEST maps with *k *= 1, 2, or 3 mismatches and genotyped using Sniper. Error bars show ± standard error of the mean over five replicates. Click here for file

Additional file 13 **Figure S8 - genotyping receiver operating characteristic curves for simulated data sets**. Receiver operating characteristic (ROC)-style curves are shown reporting SNP identification performance on four synthetic genomic DNA templates (RPL, 2 × RPL + 2%, 2 × RPL + 5%, 2 × RPL + 10%). Five unknown sample genomes were generated from each reference template by adding SNPs randomly to a proportion of 0.001. Read sets were sampled from each sample genome to one of nine coverage levels (4-fold, 10-fold, 25-fold, 32-fold, 50-fold, 75-fold, 100-fold, 150-fold, 200-fold). Read sets were independently aligned to their respective reference genome using ALL, UNI, or BEST maps with *k *= 1, 2, or 3 mismatches and genotyped using Sniper. Plots show the number of false positive SNPs plus the number of calls with no read coverage versus sensitivity. Click here for file

Additional file 14 **Figure S9 - genotyping performance for simulated data**. Estimates of true positive rate, false positive rate, and false discovery rate are provided for our four synthetic templates for all mapping strategies and mismatch conditions, based on 50× simulated read sets and genotyped using a Q ≥ 40 stringency cutoff. Estimates for the BESTNO-Q strategy (best no-guess mapping using read quality values for mapping) were based on default Bowtie settings (-n mode with -l 28 -e 70). Click here for file

Additional file 15 **Table S5 - performance estimates under variable sequencing error rates**. This Excel file provides performance estimates as described in Text S4 in Additional file 1. Click here for file

Additional file 16 **Table S6 - comparison of novel Sniper SNPs with the HapMap collection**. This Excel file contains SNP calls and statistics for a HapMap-intersecting subset of the 454 Sniper SNPs not identified by GATK and the 412 SNPs differing in genotype from GATK. Click here for file

Additional file 17 Python software implementation of Sniper (version 1.5.8). Click here for file
