# Supplementary material for: Sniper: improved SNP discovery by multiply mapping deep sequenced reads
Source: Genome Biol. 2011 Jun 20;12(6):R55. doi: 10.1186/gb-2011-12-6-r55 (PMC3218843; doi:10.1186/gb-2011-12-6-r55)
Supplement: Additional file 1 — Texts S1 to S4. Text S1: description of the analysis of repetitive elements contributing to non-unique alignments. Text S2: details of our Bayesian probability model for SNP detection. Text S3: performance estimates based on comparison to the Sanger validated data set reported in Harsimendy et al. [9]. Text S4: performance estimates obtained when varying the expected base-call sequencing error rate parameter compared to actual error rate. [file gb-2011-12-6-r55-S1.PDF]

Method

Sniper: improved SNP discovery by multiply mapping deep sequenced reads

Daniel F Simola[1,2] and Junhyong Kim[1,3]*

[1]Department of Biology, University of Pennsylvania, 433 S. University Ave, Philadelphia, PA 19104, USA

[2]Department of Cell and Developmental Biology, University of Pennsylvania, 421 Curie Blvd, Philadelphia, PA 19104, USA

[3]Penn Genome Frontiers Institute, University of Pennsylvania, 433 S. University Ave, Philadelphia, PA 19104, USA

*Correspondence: Junhyong Kim. Email: junhyong@sas.upenn.edu

Text S1. Contribution of repetitive elements to non-uniquely mappable portion of the human genome

Three lines of evidence suggest that endogenous repetitive elements may be important determinants of non-unique read mapping. First, while the number of unique alignments is not affected by template length, the number of non-unique alignments is consistently greater for shorter template lengths compared to long template lengths of the same read length, especially for short reads (Table S1 in Additional file 3). This suggests that regions of low-complexity sequence less than ~500 nt in length are not amenable to sequencing using unique reads. Second, the relative increase in read depth per locus is also template length dependent, showing the greatest increase for 250 nt templates (43.8%) and the least for 1000 nt templates

(35.3%). Third, different read lengths mostly affect the proportion of unique reads, whereas the degree of degeneracy per read is less affected (Figure S1 in Additional file 2; Table S1 in Additional file 3). These observations indicate the presence of a large class of low-complexity, interspersed <500 nt sequences—presumably Alu elements which comprise ~15% of the human genome—that in general cannot be mapped uniquely. To confirm that unique reads are disproportionately excluded from redundant sequence, we assessed the occurrence of unique reads among a set of ~10,402 evolutionarily constrained repetitive elements, representing putatively functionalized redundant sequence exapted from mobile elements [15]. While ~397,000 of the 1.04 million nt of DNA sequence (38%) are covered by at least one unique read, only 1.29 reads uniquely map to each element on average, yielding an average depth of 0.58 reads/nt compared to the expected genome-wide coverage of 7.5 reads/nt. Thus, although increasing read length and decreasing the spacing between paired-end sequences can reduce the occurrence of non-unique reads overall, a large class of functionally relevant, repetitive sequences simply may not be mappable by unique reads.

Text S2. Bayesian Probability Model for SNP detection from short sequence reads

Let G denote the resequenced genome of interest and the read set be denoted $R = \{r_1, r_2, \ldots, r_w\}$ for a total of w reads. We assume that the r_i read string might be paired or single. We define

$$P(r_i \mid s,G) \qquad (1)$$

as the probability of observing the read string r_i given that the particular substring s acted as the template with the genome G; therefore, $P(r_i \mid s,G)$ is determined by the fidelity of the sequencing chemistry. The read string r_i is possibly an imperfect copy of s and we denote $\delta(r_i,s)$ as some measure of distance between r_i and s. In the most common case, $\delta(r_i,s)$ might be the Hamming distance between the two strings r_i and s. We assume $P(r_i \mid s,G) = O(c^{-\delta(r_i,s)})$ with sufficiently large c (determined by the fidelity of the sequencing) and approximately bound the probabilities by setting $P(r_i \mid s,G) \approx 0 \; if \; \delta(r_i,s) > k$

for some fixed *k*. $P(r_i \mid s, G)$ can be modeled with a parametric probability model or in detail from Phred scores, sequence features, etc.

Given the genome of interest, *G*, we want to compute the likelihood $P(r_i \mid G)$, which can be used to computer the posterior probability of SNP at some genomic position. Let G^R be a known reference genome. To simplify computations we approximate by assuming that the difference between *G* and G^R is bounded, as discussed in more detail below. Since G^R is known, we first work towards computing $P(r_i \mid G^R)$ and then use a model of deviation of *G* from G^R to compute the conditional posterior probability $P(G \mid r_i)$.

Let

$$S^{G^R}(r_i) = \{s_0(r_i), s_1(r_i), \cdots s_d(r_i)\} \qquad (2)$$

be the set of all substrings of G^R such that $\delta(r_i, s_j) \le k \forall j$. That is, those substrings of G^R whose difference with r_i is bounded by *k*; we will informally call these the *k*-difference substrings. While the notation $S^{G^R}(r_i)$ makes explicit that this string set depends on the read string r_i, we will use the simplified notation S^{G^R} unless further clarity is needed. Since the genome is assumed to be diploid, each member of S^{G^R} may be a substring of either chromosome. We further consider the string notation to include the genomic locations of the string such that two strings s_i and s_j are distinct if they originate from different genomic locations (and chromosomes) even if their sequences are identical. We compute the conditional probability of the read string given the reference genome as,

$$\begin{aligned} P(r_i \mid G^R) &\approx P(r_i \mid S^{G^R}) \\ &= \sum_{j=1}^{d} P(r_i \mid s_j, S^{G^R}) P(s_j \mid S^{G^R}) \end{aligned} \qquad (3)$$

The first line in Eq. 3 represents the approximation that $P(r_i, G^R \mid s) \approx 0 \ if \ \delta(r_i, s) > k$. The term $P(s_j \mid S^{G^R})$ denotes the probability that the substring s_j ends up in the template pool for the genome G^R

and becomes the template for the read string r_i. This probability is determined by the efficiency at which a particular substring of the genome ends up in the template pool, which could be biased due to the template preparation chemistry. The specific bias may be modeled based on known calibration data and statistical features of the string s_j. The summation in the second line of Eq. 3 incorporates the assumption that the event that any particular substring ends up in the template pool and generates the read is disjoint. Once a string ends up in the template pool, we assume that all templates with $\delta(r_i, s) \leq k$ may act as the specific template for the read string r_i with equal probability. Thus, if no bias is present, $P(s_j \mid S^{G^R}) = 1/(d+1)$, where d+1 is $\left|S^{G^R}\right|$, the number of substrings of G^R that have k-bounded difference with r_i. This assumption is made on the grounds that the possible template strings s_j are themselves bounded by 2k differences from each other ($\delta(s_j, s_m) \leq 2k$ for $s_j, s_m \in S^{G^R}$) and are therefore unlikely to have significantly different effects on sequencing chemistry. For example, suppose k = 2 and there are two possible templates s_j and s_m for read r_i with $\delta(r_i, s_j) = 1$ and $\delta(r_i, s_m) = 2$. Assuming the same read quality scores, it is equally likely for s_j and s_m to have a single base-call error. Then given that r_i contains a true SNP (which is independent of the sequencing chemistry), s_m would be the actual template. Thus, it is important to consider all possible templates (nearly) equally. Of course, this assumption is more likely to be valid for smaller values of k.

We now need to modify Eq. 3 to compute $P(r_i \mid G)$ for the genome of interest and not the reference genome G^R. A reasonable approximation is to assume that G and G^R are sufficiently similar to each other such that if the read r_i were to be mapped to G, it would map to the same locations as those we get when we map it to G^R—this is in fact the *de facto* assumption for all resequencing studies that use alignments versus de novo assembly. We note that this assumption can be modeled more carefully by allowing the value of k, the bound on the difference, to be a function of the estimated difference between G and G^R.

Let $S^G = \{s_0, s_1, \cdots s_d\}$ be a potential k-difference substring set of G, similar to the construction in Eq. 2.

The set S^G may have many possible configurations depending on the actual genome G. We denote these possible configurations of S^G by $C(S^G)$. In the simplest case, we might assume that the sampled genome G is identical to the reference genome except at some position l. Let g_l be the genotype of position l. Then g_l has 10 possible values: AA, AC, AG….TG, TT and the number of configurations of S^G in $C(S^G)$ is 10. We might relax the assumption and consider more complex cases. Below, we describe an intermediate model that was implemented in our program, Sniper, where we assumed that the sampled genome G is such that each substring s_i of S^G is at most 1 SNP different from the corresponding paralogous positions of the substrings in the reference genome. For example, if s_0 has a SNP in the kth position, no other string s_1–s_d has a SNP in the same position. This is equivalent to saying that paralogous substrings do not have shared SNPs.

For SNP calling, we will be interested in the marginal case focused on the genotype of g_l regardless of the other genomic locations. Without loss of generality we assume that position l of interest in the resequenced genome G is covered by the subsequence s_0 of S^G. To obtain the string set, S^G, for the resequenced genome, we consider the expansion of s_0 to include the possible variant genotypes at position l. Let s_0^A denote the variant subsequence of s_0 whose nucleotide letter in the position corresponding to genomic position l is A, and so on. Let $G_{xy(l)}$ be a possible sampled genome with genotype xy at position l, and denote the k-difference substring set $S_{xy(l)}^G = \{s_0^x, s_0^y, s_1, \cdots s_d\}$ for the read r_i. Then

$$\begin{aligned} P(r_i \mid G_{xy(l)}) &\approx P(r_i \mid S^G_{xy(l)}) \\ &= P(r_i \mid s_0^x, S^G_{xy(l)}) P_{r_i}(s_0^x \mid S^G_{xy(l)}) + P(r_i \mid s_0^y, S^G_{xy(l)}) P_{r_i}(s_0^y \mid S^G_{xy(l)}) + 2\sum_{j=1}^{d} \max_X \{P(r_i \mid s_j^X, S^G_{xy(l)}) P_{r_i}(s_j^X \mid S^G_{xy(l)})\} \end{aligned} \quad (4)$$

where the last term denotes the conservative assumption to bound the paralogous loci likelihood at the maximum of possible nucleotides, i.e., $X \in \{A, T, C, G\}$. Eq. 4 considers the possibility that the genotypes of the paralogous loci of l may differ from those indicated by the reference genome G^R.

We assume that given a genome the template preparations and subsequent sequencing chemistries do not interact for different reads. Therefore, we assume that

$$P(r_0, r_1, \cdots, r_n \mid G_{xy(l)}) = n! \prod P(r_i \mid G_{xy(l)}) \approx n! \prod P(r_i \mid S^G_{xy(l)}(r_i)) \tag{5}$$

where the term $S^G_{xy(l)}(r_i)$ indicates that the k-difference substring set for each read r_i can be different. We note that in some empirical situations the independence assumption encapsulated in Eq. 5 may not be true (*e.g.,* if total template molecules are limiting, it can induce negative relationships between different reads).

Using the standard Bayesian inversion formula we have:

$$\begin{aligned} P(G_{xy(l)} \mid r_0, r_1, \cdots r_n) &= \frac{P(r_0, r_1, \cdots r_n \mid G_{xy(l)}) P(G_{xy(l)})}{\sum_{XY} P(r_0, r_1, \cdots r_n \mid G_{XY(l)}) P(G_{XY(l)})} \\ &= \frac{\left[\prod_i P(r_i \mid G_{xy(l)})\right] P(G_{xy(l)})}{\sum_{XY} \left[\left[\prod_i P(r_i \mid G_{XY(l)})\right] P(G_{XY(l)})\right]} \\ &\approx \frac{\left[\prod_i P(r_i \mid S^G{}_{xy(l)}(r_i))\right] P(S^G{}_{xy(l)}(r_1) \cdots S^G{}_{xy(l)}(r_n))}{\sum_{XY} \left[\left[\prod_i P(r_i \mid S^G{}_{XY(l)}(r_i))\right] P(S^G{}_{XY(l)}(r_1) \cdots S^G{}_{XY(l)}(r_n))\right]} \end{aligned} \tag{6}$$

where the denominator sums over all possible genotypes $xy \in XY = \{AA, AC, \cdots TT\}$ at position l of interest.

We first note that if a read string does not cover site l (and its related k-difference genomic substrings) within the predefined k distance, its probability is invariant with respect to the genotype at l and contributes a constant factor to both numerator and denominator of Eq. 6. Therefore, for calculations at site l, we can ignore all reads that do not cover site l within k difference. Thus the formula is applied over only the subset of reads that align to position of interest—here we assume we have n such reads.

The term $P(S^G_{xy(l)}(r_1)\cdots S^G_{xy(l)}(r_n))$ represents the joint prior probability of drawing the *k*-difference set $S^G_{xy(l)}(r_i)$ for each read r_i. Since the *k*-difference sets for each read is assumed to be derived from substrings of a possible genome $G_{xy(l)}$, each *k*-difference set is not independent of each other and the joint prior cannot be computed from products. To compute the joint prior we first delimit the genome into independent intervals that are relevant to the substrings of $S^G_{xy(l)}(r_i)$ for $\forall\, i$ and then consider the possible sequence variants of each of the independent intervals. Recall that $S^G_{xy(l)}(r_i) = \{s^x_0(r_i), s^y_0(r_i), s_1(r_i), \cdots s_d(r_i)\}$. That is, each set $S^G_{xy(l)}(r_i)$ is a collection of genomic substrings, such that we have a collection of genomic substrings for each read string r_i. We concatenate the genomic substrings in all $S^G_{xy(l)}(r_i)$ such that if two substrings for two different reads overlap in their genomic location the two strings are merged into the longer substring. That is, if $s_a(r_i) \cap s_b(r_j) \neq \phi$ (in genomic location) for any *a, b, i, j*, we create a new string $t_k = s_a(r_i) \cdot s_b(r_j)$. Using this procedure we create the genomic substring set $T^G_{xy(l)} = \{t^x_0, t^y_0, \cdots t_g\}$, whose members are disjoint intervals of the genomic sequence. Then

$$P(S^G_{xy(l)}(r_1)\cdots S^G_{xy(l)}(r_n)) = P(S^G_{xy(l)}) \text{ such that } \forall s \in S^G_{xy(l)}(r_i),\, s \subseteq t \text{ for some } t \in T^G_{xy(l)} \quad (7)$$

That is, the joint prior probability of the sets of *k*-difference substrings is the probability of the genomic string configuration that implies each of the substrings.

We can now create a model of possible genomic variation over these disjoint intervals similar to the discussion above on possible configurations for any individual $S^G_{xy(l)}(r_i)$. In particular, a prior probability model of drawing a genome with genotype *xy* at any variant position might be modulated by a model assumption on population-level heterozygosity and potentially the transition probability of any mutant allele types. One possibility is to segregate the genotypes into four categories: homozygous identical to reference genome (h0); heterozygous with one allele different from the reference genome (t1); homozygous

with both alleles different from the reference genome (h2); and heterozygous with both alleles different from the reference genome (t2). The notation refers to h = homozygous, t = heterozygous; 0, 1, 2, number of alleles different from reference. We denote p_{h0}, p_{t1}, p_{h2}, p_{t2} for the respective probabilities. Probabilities p_{h2} and p_{t2} can be different because homozygous genotypes whose both alleles are different from the reference can arise out of a single mutation and then inbreeding, while heterozygous, two-allele differences require two mutations. As mentioned before, in the simplest model we only consider the configurations where there is potential for a single SNP variation at position *l*. In the more general models, we can compute the joint probability in Eq. 7 by taking the product of p_{h0}, p_{t1}, p_{h2}, p_{t2} terms over each independent genomic interval, respecting the corresponding variant configuration.

Lastly, we are interested in $P(g_l = xy \mid r_1 \cdots r_n)$, the marginal posterior probability that the genotype at g_l locus is *xy* given the *n* reads. From (6) we have

$$P(g_l = xy \mid r_1 \cdots r_n) \approx \sum_{C(T^G_{xy(l)})} P(T^G_{xy(l)} \mid r_1 \cdots r_n) \qquad (8)$$

where the summation is over the possible configuration set $C(T^G_{xy(l)})$ of the genomic string set. In our program, we implemented a case analysis of having at most one SNP at paralogous loci over the aligned substrings of the genome. Let, g_0 be the genotype at the locus of interest and g_1 to g_d denote the genotype at the paralogous positions in the other alignments. Let the nucleotide identity for the read r_i at the position of interest is either *R* or *D*, where *R* is the reference genome allele type at g_0 and *D* is a derived allele type at g_0 (we assume *D* represents any of 3 possible derived alleles). The marginal probability of the genotype at g_0 will be dominated by the probability of *R* or *D* alleles generated by g_1 to g_d. Therefore, we can concentrate on the cases of g_j = *RR* or *RD* or *DD*. For simplicity, we also assume that the *DD* genotype is much less probable than the *RD* genotype at any locus. The joint genotypes of interest then reduce to:

$$
\begin{aligned}
&case\,0:(g_0 = RD, g_1 = RR, ..., g_d = RR)\\
&case\,1:(g_0 = RR, g_1 = RD, ..., g_d = RR)\\
&\vdots\\
&case\,d:(g_0 = RR, g_1 = RR, ..., g_d = RD)\\
&case\,d+1:(g_0 = RR, g_1 = RR, ..., g_d = RR)
\end{aligned}
\qquad (9)
$$

For each of these cases, we can compute a posterior probability given by Eqs. 4 and 5. Case 0 will yield the posterior probability of a SNP at locus of interest g_0 while the sum over cases 1 to d+1 will yield the posterior probability of a non-SNP at g_0 (*i.e.* $g_0 = RR$). Using this Bayesian scheme, we can set a threshold defining the minimum posterior probability for accepting a SNP as significant. This threshold can be interpreted as a stringency parameter.

The above probability model then suggests an algorithm for Bayesian estimation of genotypes as follows. (1) For sequencing read set R, map all reads to a reference genome up to *k* mismatches, including those that map to multiple genomic locations. (2) Let *l* be a genomic position of interest. Assemble all reads that overlap position *l* and also note any other position on the genome that maps to each read to construct the sets $S^G(r_i)$ for each read r_i that overlap *l*. (3) Generate a genomic string set T^G by concatenating all the alignments over each read. (4) Generate possible variant sets $T^G_{xy(l)}$ by considering the possible genotype variations at position *l* (as well as the other positions). (5) Use Eqs. 4, 6, and 8, iterating over the possible genotypes at *l*, the paralogous aligned loci, and their respective prior probabilities.

Text S3. Performance validation using ABI Sanger verified loci

To validate the performance of our method, called Sniper (SNP Identification using the Probability of Every Read), we estimated genotypes for 261 kb of the human genome for 4 individuals using 36 nt Illumina 1G single-end read data, previously described in Harsimendy et al. (2009) [9]. To assess how SNP calling differs across mapping strategies, we computed genotypes using unique (UNI), best-guess (BEST), and total max-*d* (ALL) read mapping strategies (see Figure 1B). We also compared our method to Maq [4]

and SOAPsnp [5], two alternative methods that generate SNP calls using either unique or best-guess strategies. In our experiments UNI and BEST are specified by a single parameter *k*, whereas ALL is specified by 2 parameters, *k* and *d*. We tested $k = 1, 2$, and 3 for UNI, BEST, and ALL, with $d+1 = 200$, which is approximately the sequencing coverage of our data (~188-fold). (While choice of *d* showed little overall affect on performance on this data set [data not shown], in general increasing *d* improves the approximation to the true read map, up to a point—see Eq. 9 in main text) To control for performance variation due to the mapping algorithm, identical read maps, generated by Bowtie [27], were used as input for both Sniper and Maq; due to technical issues, the BEST map for SOAPsnp was generated using SOAPalign [5]. We note that our comparison of ALL, UNI, and BEST maps through Sniper fully controls for performance variation due to software implementation. Thus, performance estimates should directly reflect the quality of the read map approximation.

First, we compared significant SNP predictions generated by Sniper, Maq, and SOAPsnp to SNP calls generated by ABI Sanger sequencing to estimate true positive rate (TPR) and false discovery rate (FDR) for each method. Averaging over results from the 9 different mismatch and read map combinations for Sniper, we estimate that the overall FDR of our method is 1.43% and the TPR is 81.49% using a lenient per-locus posterior probability error threshold (stringency) of $P < 0.05$ ($Q \geq 13$). These estimates are stable as stringency increases to $P < 10^{-9}$ ($Q \geq 90$) (Table S3 in Additional file 8). The optimal TPR and FDR of 83.1% and 1.37%, respectively, are found for $k = 2$ and BEST. These estimates are comparable to the best results estimated using Maq (TPR=83.4%, FDR=1.35%) and SOAPsnp (TPR=84.01%, FDR=1.36%) (Table S3 in Additional file 8).

While these TP rates may seem low, the majority of SNP loci identified by Sanger sequencing but not by Sniper (55 putative false negatives for $k = 2$ mismatches and BEST) are not predictable from this data set due to lack of sufficient sequencing coverage; 20 loci overlap with at most five reads per locus, while another 34 loci overlap with at most five reads that support the variant (non-reference) allele. Thus despite

sequencing this 261 kb region to near-saturating coverage, several loci nevertheless lack sufficient coverage for accurate SNP discovery, likely due to severe bias in genomic DNA library preparation or sequencing. Since only 1 out of 55 false negative loci have sufficient coverage to detect a polymorphism by our method, our reported TP rates are conservative and appropriate for the dataset. Furthermore, all nine conditions tested using Sniper, in addition to most conditions for Maq and SOAPsnp, predict a common set of four SNPs that are not predicted by the Sanger genotypes. Each of these four SNPs shows high coverage (depth of 53–395 reads), low error probability ($P < 10^{-16}$), and estimated minor allele frequencies close to 0.5 (Table S4 in Additional file 14). Thus, we expect that these four loci in fact harbor true polymorphisms that were not discovered by Sanger sequencing.

Since our TP and FP estimates appear to be conservative, we also compared our results to a subset of 253 of the 344 loci that were identified as variant by three different NGS platforms [9] and which inferred identical genotypes in agreement with ABI Sanger sequencing. For our method, this comparison yields an estimated TPR of 97.2% and an estimated FDR of 0.0%, averaged over the nine mismatches and read map combinations (Table S3 in Additional file 8). The combination of $k = 2$ mismatches and BEST again exhibits maximal TPR and FDR of 99.2% and 0.0% respectively, comparable to Maq (TPR=99.6%, FDR=0.0%) and SOAPsnp (TPR=98.8%, FDR=0.0%). Thus, comparison of performance metrics based on Harsimendy et al. Sanger verified SNP loci clearly demonstrates that our method performs comparably to existing methods.

Text S4. False discovery rates remain low using multiply mapped reads for variable sequencing error rates

Our Bayesian model is parameterized by the expected global sequencing error rate for a next-generation sequencing platform, e. The value of e may differ from the true (unknown) error rate, ε, for a given experiment, and this difference could greatly affect false positive and false negative error rates. To evaluate whether using multiply mapped reads helps to maintain a low error rate when $e \neq \varepsilon$, as may occur when

analyzing experimental data, we generated SNP calls after setting e to be ~10-fold lower (more lenient) or ~10-fold higher (more conservative) than ε, using the 2x RPL +5% template and simulated reads described above (where $\varepsilon \approx \pi = 0.001$, the expected sequence divergence). As expected, setting $e >> \varepsilon$ increases false negative rate while maintaining a false positive error rate of 0.0, and setting $e << \varepsilon$ decreases in false negative rate while increasing false positive rate slightly, for ALL, UNI, and BEST (Table S5 in Additional file 15). Overestimating the true error rate ($e << \varepsilon$), however, results in an increased false discovery rate from 5.7% to 11% for BEST, but only rises to 2.5% for ALL (from 0.078%). This change in e represents a 1.9-fold increase in FDR for BEST, a 3.2-fold increase for ALL, and a 6.6-fold increase for UNI; while in this situation ALL increases more quickly than BEST, its estimated FDR rate remains 4.4-fold lower than that of BEST. Despite this low FDR, ALL also shows the same sensitivity as BEST (TPR = 83.3%). In addition, genotyping with $e = 1 \times 10^{-5}$ (100-fold lower than ε) only increases the FDR of ALL to 6.8% (19.5% for BEST; 3.8% for UNI). We also tested whether our method remains conservative if the true error rate exceeds the SNP rate, since $\varepsilon \approx \pi$ for previous results. We simulated 5 replicate sequence read sets at 50-fold coverage with $\varepsilon = 0.01$, which is 10-fold higher than π. While all three maps show a large decrease in sensitivity (from ~80% to ~17%), their false positive rate remains 0.0. Thus our method exhibits two desirable properties: (1) SNP calling remains conservative when underestimating the true sequencing error rate ($e >> \varepsilon$); and (2) the false SNP discovery rate remains small when drastically overestimating the error rate ($e << \varepsilon$). Using multiply mapped reads may be critical for maintaining a low false discovery rate on real data, especially those with longer sequence reads, which can exhibit large fluctuations in sequencing error rates due to incorrect image phasing.
