# Supplementary material for: Sniper: improved SNP discovery by multiply mapping deep sequenced reads
Source: Genome Biol. 2011 Jun 20;12(6):R55. doi: 10.1186/gb-2011-12-6-r55 (PMC3218843; doi:10.1186/gb-2011-12-6-r55)
Supplement: Additional file 2 — Figure S1 - simulated paired-end read multiplicity distributions for the human genome. The number of valid alignments against the Homo sapiens genome is reported as a proportion of the 2 × 10^6^ randomly sampled PE reads used in alignment, varying fragment length (250, 500, 750, or 1,000 nucleotides) and read length (30, 60, 90, or 120 nucleotides). The proportion of read multiplicity averaged over reads of the same length is shown in each figure. [file gb-2011-12-6-r55-S2.PDF]

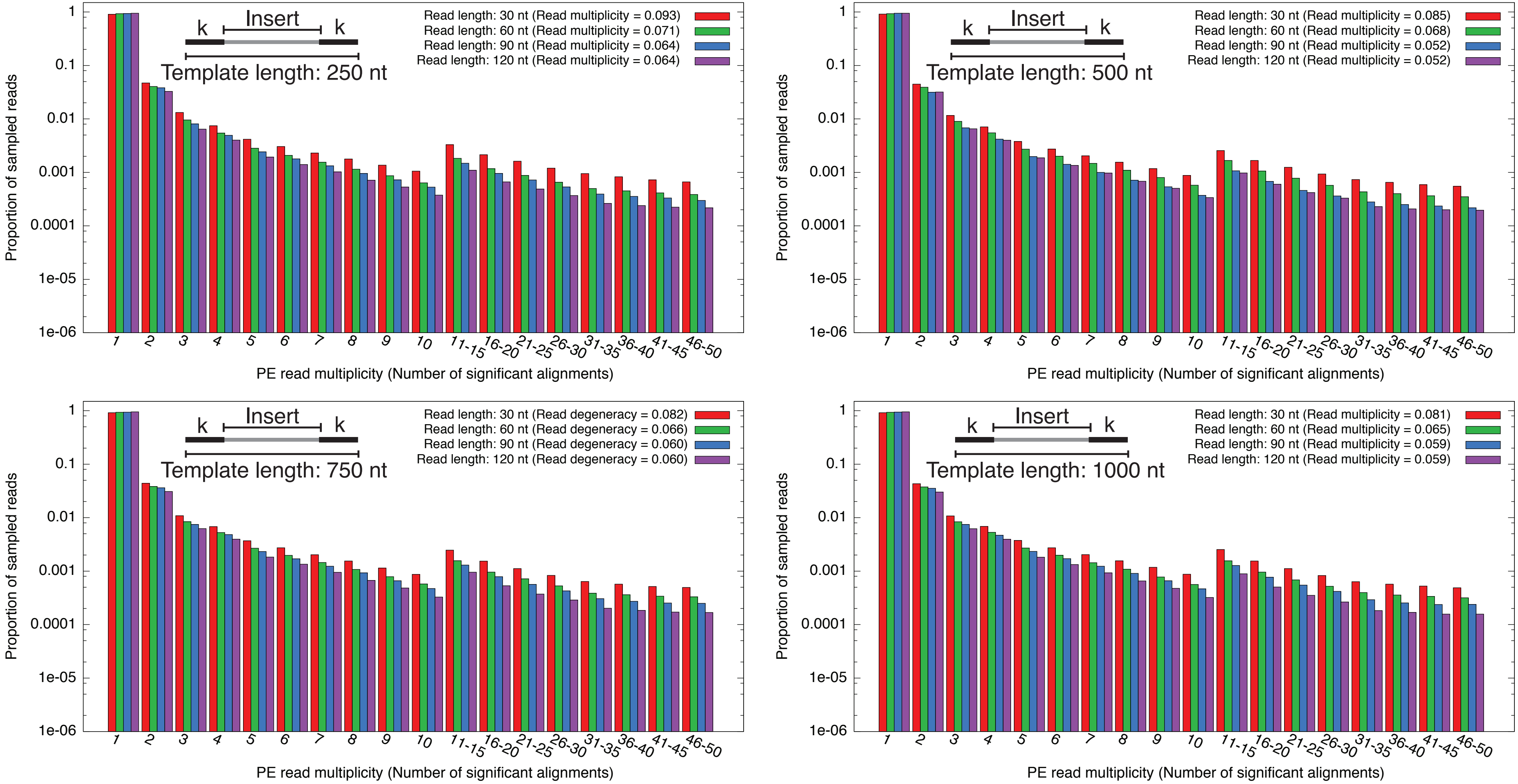

Insert
k
k
Template length: 250 nt
Read length: 30 nt (Read multiplicity = 0.093)
Read length: 60 nt (Read multiplicity = 0.071)
Read length: 90 nt (Read multiplicity = 0.064)
Read length: 120 nt (Read multiplicity = 0.064)
Template length: 500 nt
Read length: 30 nt (Read multiplicity = 0.085)
Read length: 60 nt (Read multiplicity = 0.068)
Read length: 90 nt (Read multiplicity = 0.052)
Read length: 120 nt (Read multiplicity = 0.052)
Template length: 750 nt
Read length: 30 nt (Read degeneracy = 0.082)
Read length: 60 nt (Read degeneracy = 0.066)
Read length: 90 nt (Read degeneracy = 0.060)
Read length: 120 nt (Read degeneracy = 0.060)
Template length: 1000 nt
Read length: 30 nt (Read multiplicity = 0.081)
Read length: 60 nt (Read multiplicity = 0.065)
Read length: 90 nt (Read multiplicity = 0.059)
Read length: 120 nt (Read multiplicity = 0.059)
Proportion of sampled reads
1
0.1
0.01
0.001
0.0001
1e-05
1e-06
1 2 3 4 5 6 7 8 9 10 11-15 16-20 21-25 26-30 31-35 36-40 41-45 46-50
PE read multiplicity (Number of significant alignments)
