# Supplementary figures and images for: Sniper: improved SNP discovery by multiply mapping deep sequenced reads
Source: Genome Biol. 2011 Jun 20;12(6):R55. doi: 10.1186/gb-2011-12-6-r55 (PMC3218843; doi:10.1186/gb-2011-12-6-r55)

## A False mapping due to SNP

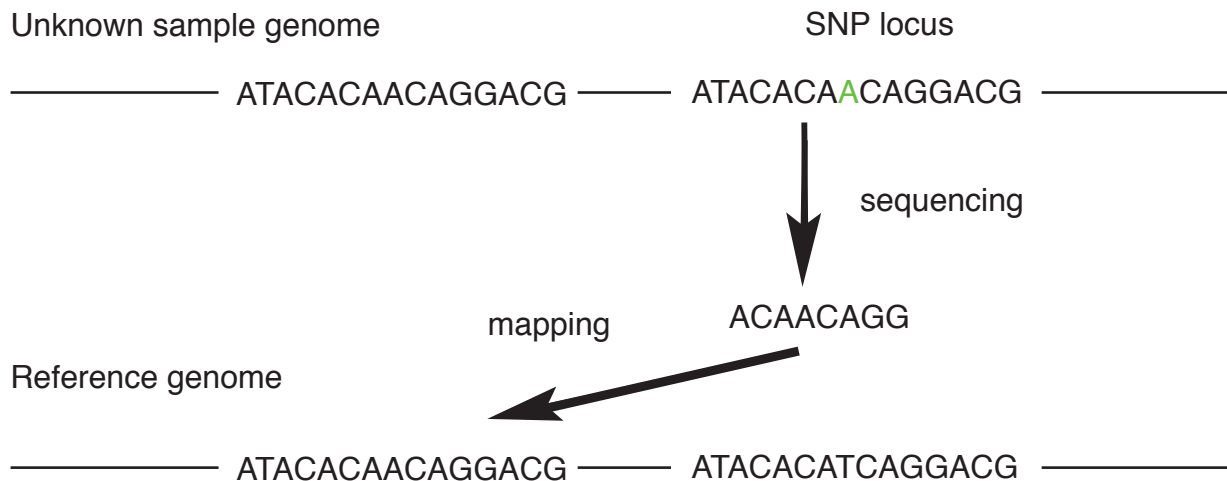

## B False mapping due to base-call error

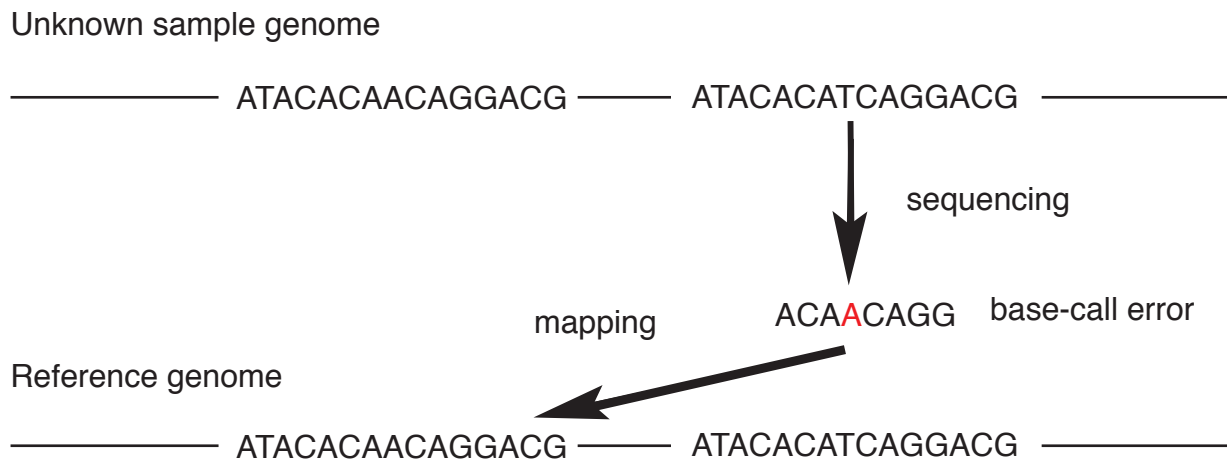

Supplement: Additional file 4 — Figure S2 - examples of false read mapping. False read mapping occurs when a sequenced read is incorrectly aligned to its reference sequence (that is, to the incorrect location in the genome). This is most likely to occur in the presence of closely related sequences existing in replicate in the reference sequence and can result from either (a) SNP occurrence or (b) base-call sequencing error in the sample genome, such that the similarity between reads containing a variant (or false) allele and the reference genome decreases at one locus and increases at another (false) locus. Instances of false mapping consequently decrease the chance of a SNP call at a true locus and increase the chance of a false SNP call at the wrong locus. [file gb-2011-12-6-r55-S4.PDF]

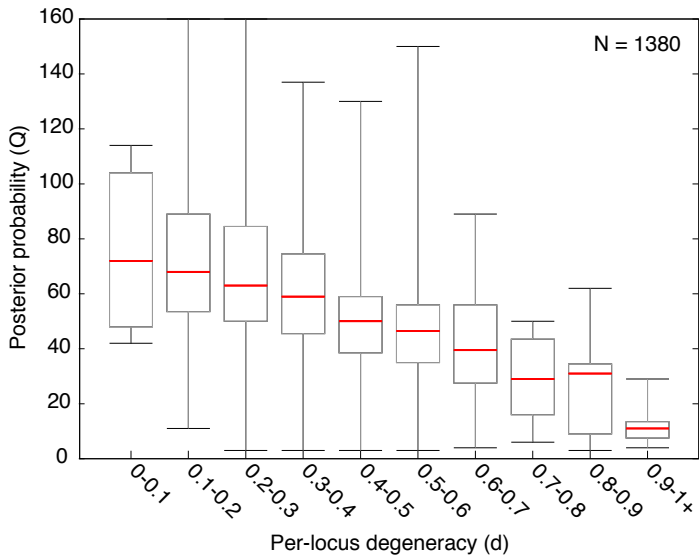

Supplement: Additional file 6 — Figure S3 - relationship between posterior probability and read degeneracy. Box and whisker plots showing the distribution of posterior probabilities Q (stringency) for all SNPs identified in each of five replicates for two different simulations (ribosomal protein loci (RPL) and 2 × RPL + 10%), grouped by per-locus degeneracy. For example, the 0 ≤ <d < 0.1 group contains loci with at least a 1/10 ratio of alignments at another locus versus alignments overlapping the locus of interest. Box plots represent the entire distribution of Q values for each degeneracy bin, where the red line indicates median and the box indicates the 25th and 75th percentiles. SNPs were obtained using the 25-fold coverage simulations allowing k ≤ 1 mismatch. [file gb-2011-12-6-r55-S6.PDF]

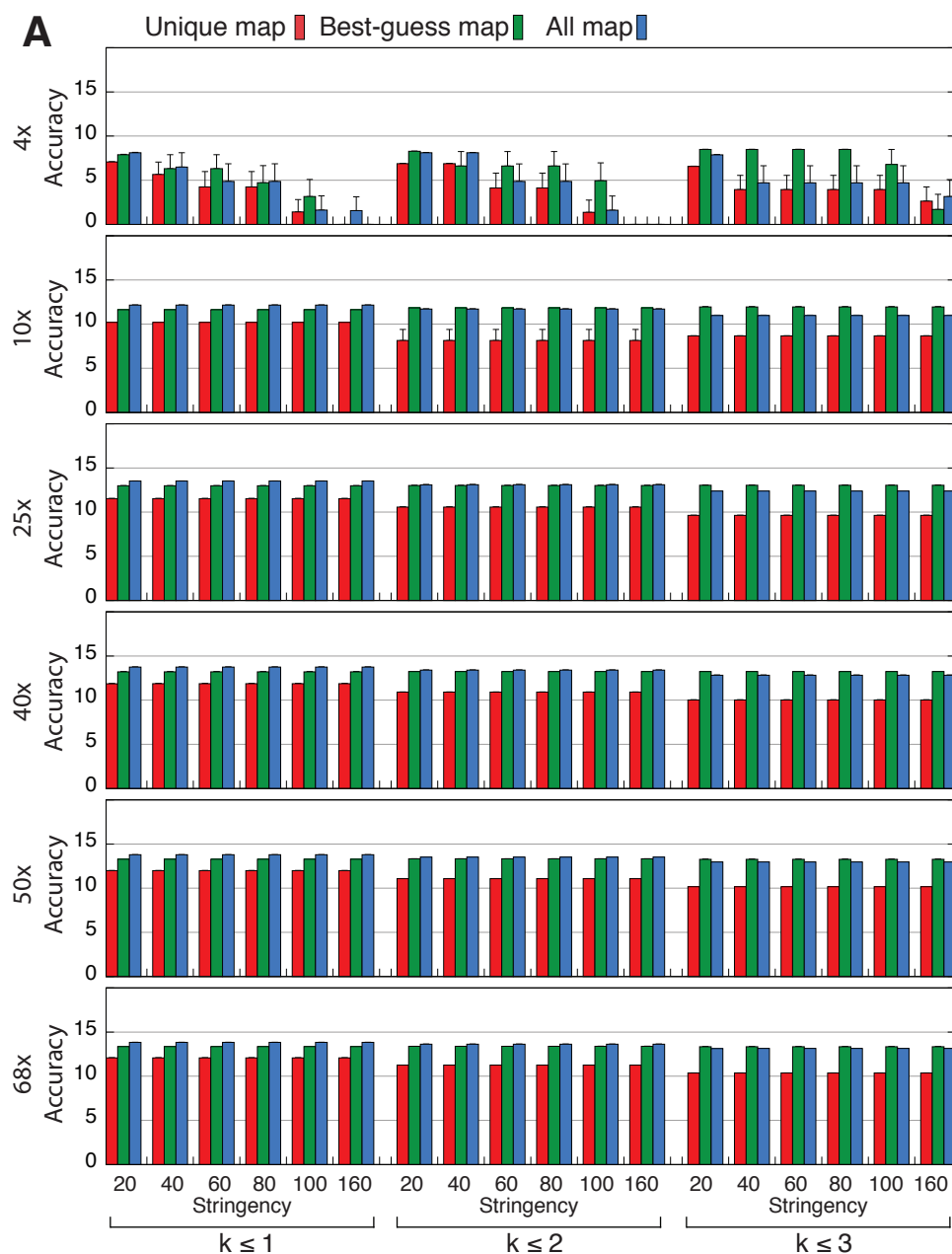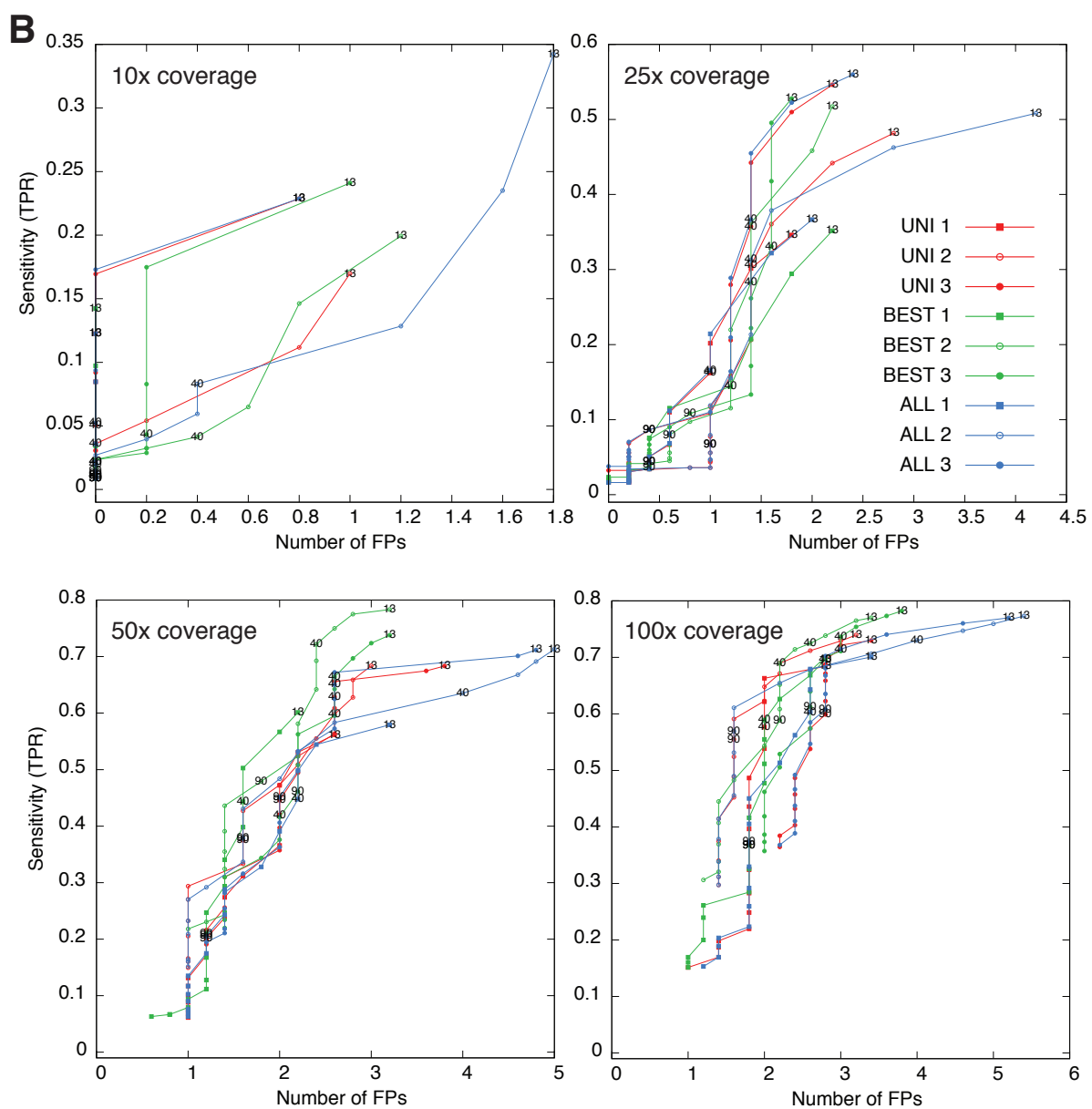

Supplement: Additional file 10 — Figure S5 - human genotyping performance across coverage levels. Accuracy bar charts and Receiver operating characteristic (ROC)-style curves for human SNP identification at six coverage levels. Reads from one individual (NA17156) were subsampled randomly from the complete approximately 188-fold coverage data in five replicates. Each subsampled read set was independently aligned to the human genome using ALL, UNI, or BEST maps with k = 1, 2, or 3 mismatches and genotyped using Sniper. (a) Bar charts reporting genotyping accuracy for each condition. Error bars show ± standard error of the mean. (b) ROC-style curves are shown as 1 - accuracy versus sensitivity. Three stringency levels (Q ≥ 13, 40, 90) are shown for each curve. [file gb-2011-12-6-r55-S10.PDF]

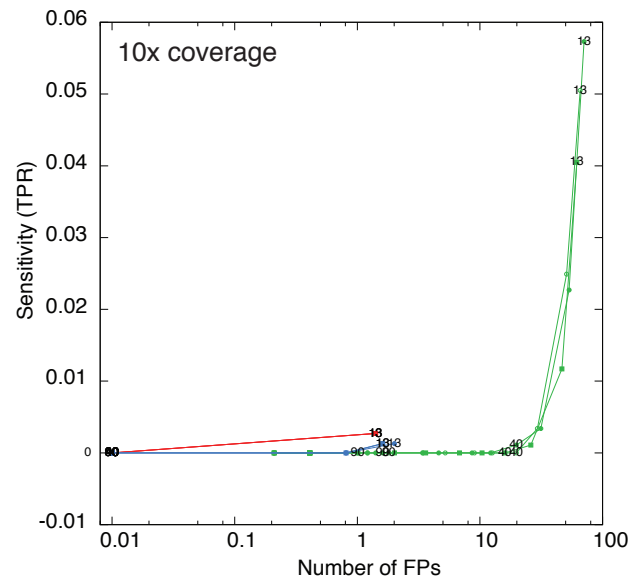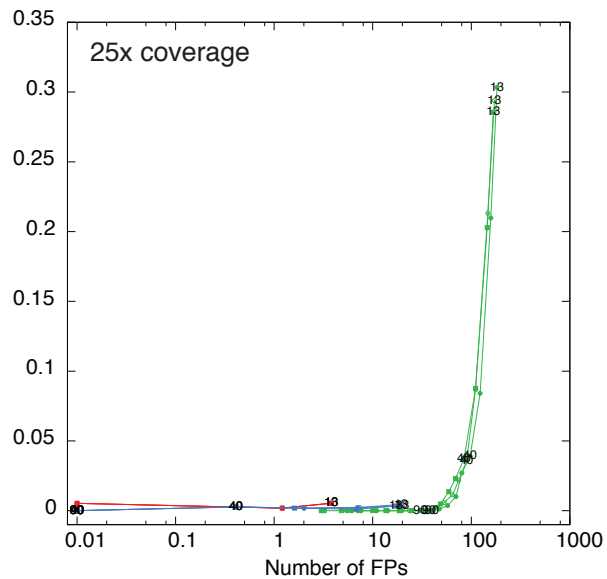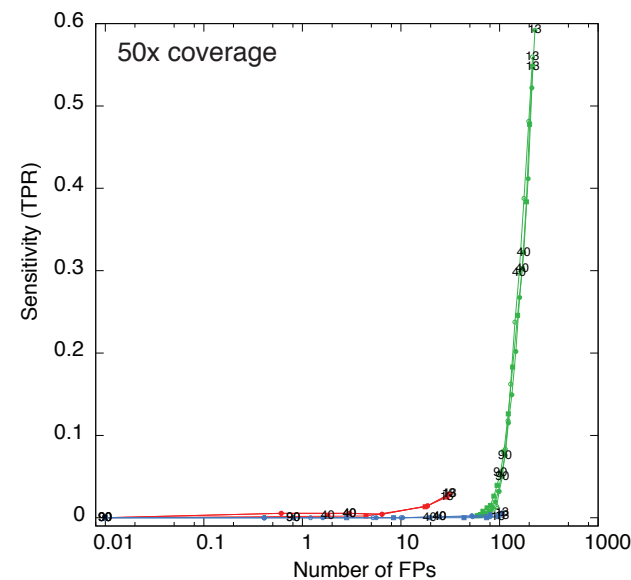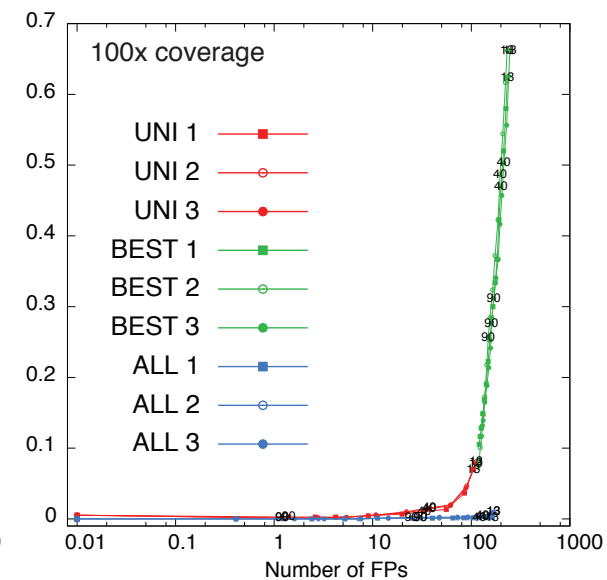

Supplement: Additional file 11 — Figure S6 - simulation negative control. (a) Accuracy bar charts and (b) ROC-style curves showing performance of ALL, UNI, and BEST maps on our negative control synthetic DNA template (2 × RPL +0%). Five unknown sample genomes were generated from each reference template by adding SNPs randomly to a proportion of 0.001. Read sets were sampled from each sample genome to one of four coverage levels (25-fold, 50-fold, 100-fold, 200-fold). Read sets were independently aligned to their respective reference genome using ALL, UNI, or BEST maps with k = 1, 2, or 3 mismatches and genotyped using Sniper. (a) Bar charts report genotyping accuracy for each condition. Error bars show ± standard error of the mean over five replicates. (b) ROC-style curves are shown as the number of false positive calls versus sensitivity. Three stringency levels for Q (13, 40, 90) are shown for each curve. [file gb-2011-12-6-r55-S11.PDF]

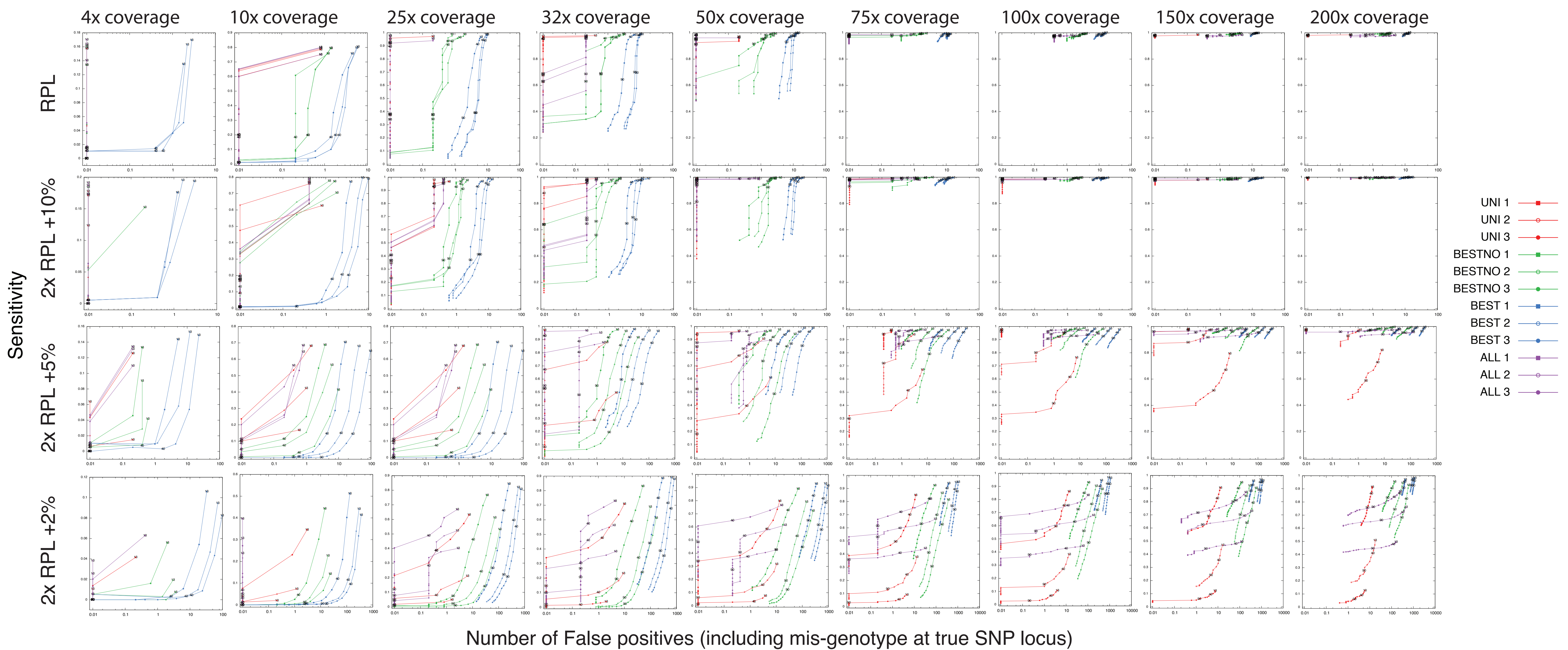

Supplement: Additional file 13 — Figure S8 - genotyping receiver operating characteristic curves for simulated data sets. Receiver operating characteristic (ROC)-style curves are shown reporting SNP identification performance on four synthetic genomic DNA templates (RPL, 2 × RPL + 2%, 2 × RPL + 5%, 2 × RPL + 10%). Five unknown sample genomes were generated from each reference template by adding SNPs randomly to a proportion of 0.001. Read sets were sampled from each sample genome to one of nine coverage levels (4-fold, 10-fold, 25-fold, 32-fold, 50-fold, 75-fold, 100-fold, 150-fold, 200-fold). Read sets were independently aligned to their respective reference genome using ALL, UNI, or BEST maps with k = 1, 2, or 3 mismatches and genotyped using Sniper. Plots show the number of false positive SNPs plus the number of calls with no read coverage versus sensitivity. [file gb-2011-12-6-r55-S13.PDF]

RPL  
RPL +10%  
2x RPL +5%  
2x RPL +2%

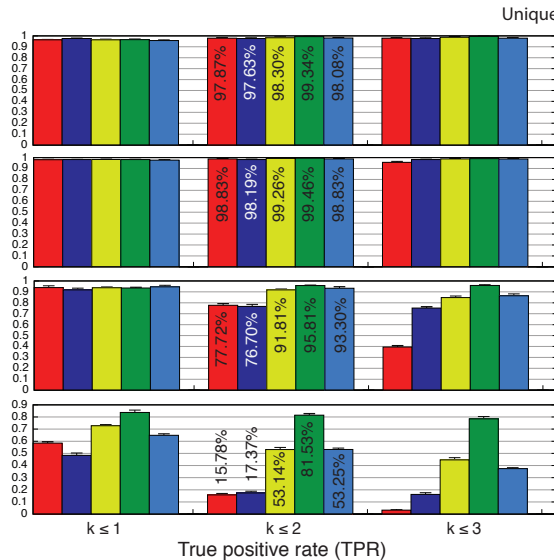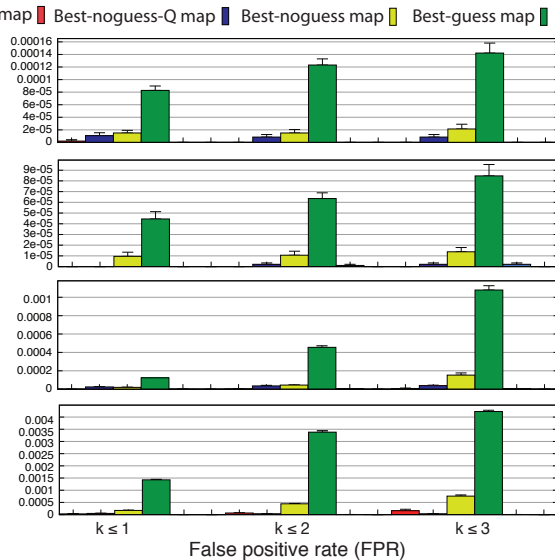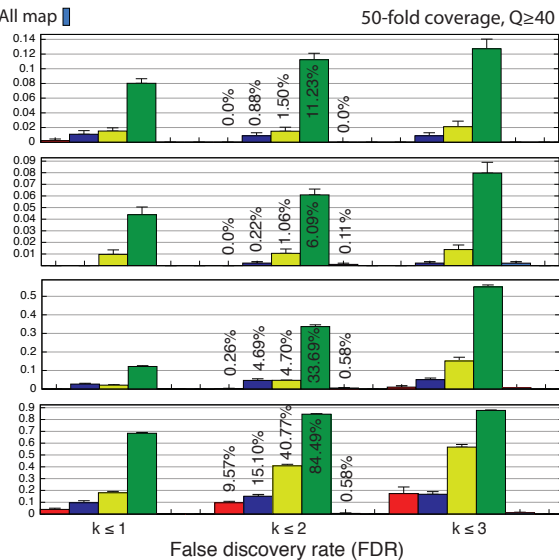

Supplement: Additional file 14 — Figure S9 - genotyping performance for simulated data. Estimates of true positive rate, false positive rate, and false discovery rate are provided for our four synthetic templates for all mapping strategies and mismatch conditions, based on 50× simulated read sets and genotyped using a Q ≥ 40 stringency cutoff. Estimates for the BESTNO-Q strategy (best no-guess mapping using read quality values for mapping) were based on default Bowtie settings (-n mode with -l 28 -e 70). [file gb-2011-12-6-r55-S14.PDF]
