# Supplementary material for: Sniper: improved SNP discovery by multiply mapping deep sequenced reads
Source: Genome Biol. 2011 Jun 20;12(6):R55. doi: 10.1186/gb-2011-12-6-r55 (PMC3218843; doi:10.1186/gb-2011-12-6-r55)
Supplement: Additional file 7 — Figure S4 - per-locus distributions of degeneracy. Cumulative distributions of per-locus degeneracy are shown for each of the six reference genomic DNA templates used in this study. Degeneracy is defined as the ratio of d, the number of alignments for a read that overlap loci other than the locus of interest, to the read depth at a locus of interest. (Alternatively, Alignments/Reads - 1.) For example, a ratio of 1 indicates that every read overlapping the locus of interest has two valid alignments in the reference genome. Loci are binned into 12 groups and the cumulative frequency of all loci is reported at each degeneracy group. Estimates are shown using the ALL read map with k = 1, 2, and 3 mismatches. [file gb-2011-12-6-r55-S7.PDF]

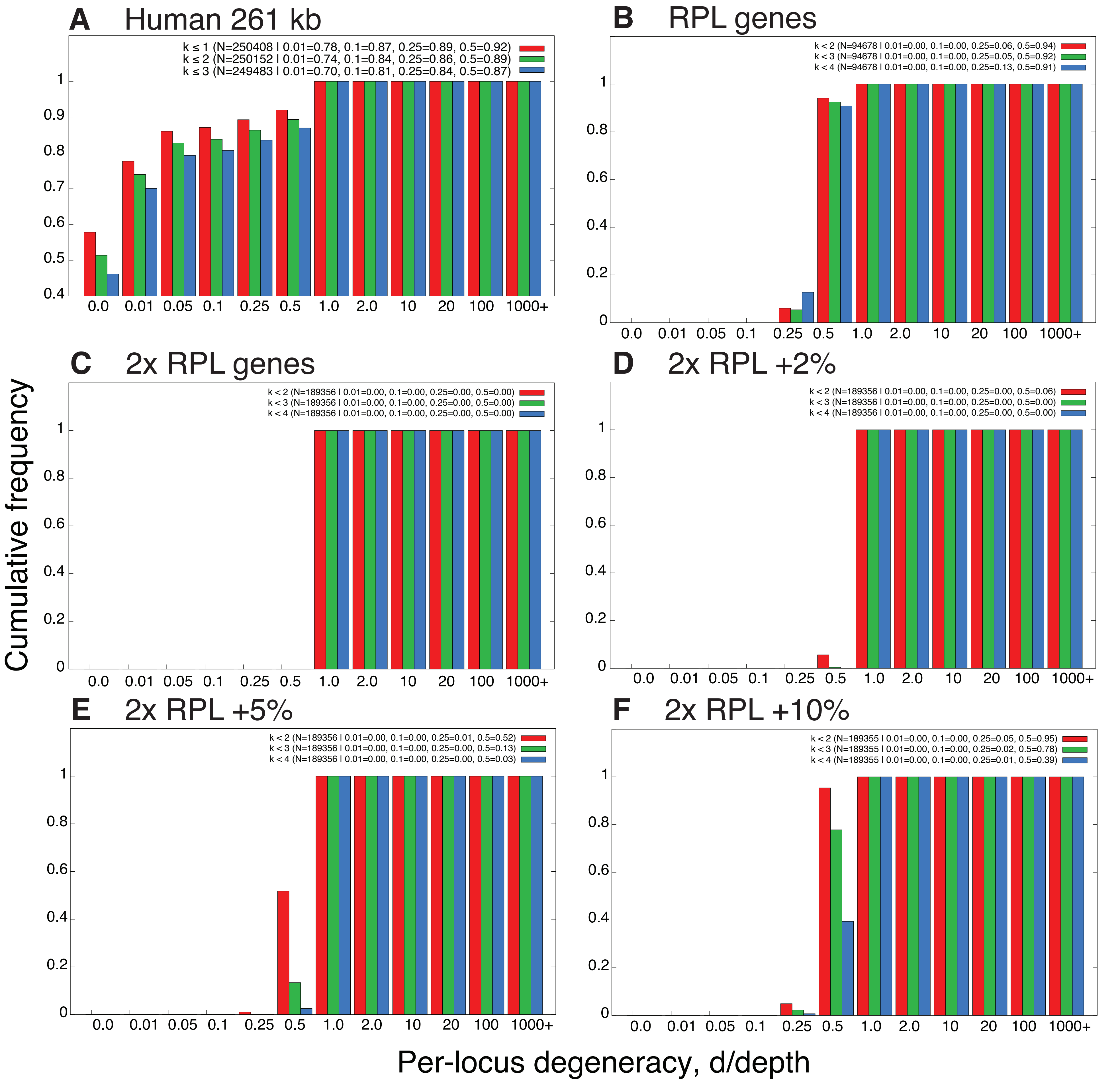

A Human 261 kb
k ≤ 1 (N=250408 | 0.01=0.78, 0.1=0.87, 0.25=0.89, 0.5=0.92)
k ≤ 2 (N=250152 | 0.01=0.74, 0.1=0.84, 0.25=0.86, 0.5=0.89)
k ≤ 3 (N=249483 | 0.01=0.70, 0.1=0.81, 0.25=0.84, 0.5=0.87)
B RPL genes
k < 2 (N=94678 | 0.01=0.00, 0.1=0.00, 0.25=0.06, 0.5=0.94)
k < 3 (N=94678 | 0.01=0.00, 0.1=0.00, 0.25=0.05, 0.5=0.92)
k < 4 (N=94678 | 0.01=0.00, 0.1=0.00, 0.25=0.13, 0.5=0.91)
C 2x RPL genes
k < 2 (N=189356 | 0.01=0.00, 0.1=0.00, 0.25=0.00, 0.5=0.00)
k < 3 (N=189356 | 0.01=0.00, 0.1=0.00, 0.25=0.00, 0.5=0.00)
k < 4 (N=189356 | 0.01=0.00, 0.1=0.00, 0.25=0.00, 0.5=0.00)
D 2x RPL +2%
k < 2 (N=189356 | 0.01=0.00, 0.1=0.00, 0.25=0.00, 0.5=0.06)
k < 3 (N=189356 | 0.01=0.00, 0.1=0.00, 0.25=0.00, 0.5=0.00)
k < 4 (N=189356 | 0.01=0.00, 0.1=0.00, 0.25=0.00, 0.5=0.00)
E 2x RPL +5%
k < 2 (N=189356 | 0.01=0.00, 0.1=0.00, 0.25=0.01, 0.5=0.52)
k < 3 (N=189356 | 0.01=0.00, 0.1=0.00, 0.25=0.00, 0.5=0.13)
k < 4 (N=189356 | 0.01=0.00, 0.1=0.00, 0.25=0.00, 0.5=0.03)
F 2x RPL +10%
k < 2 (N=189355 | 0.01=0.00, 0.1=0.00, 0.25=0.05, 0.5=0.95)
k < 3 (N=189355 | 0.01=0.00, 0.1=0.00, 0.25=0.02, 0.5=0.78)
k < 4 (N=189355 | 0.01=0.00, 0.1=0.00, 0.25=0.01, 0.5=0.39)
0.0 0.01 0.05 0.1 0.25 0.5 1.0 2.0 10 20 100 1000+
Cumulative frequency
Per-locus degeneracy, d/depth
