# Supplementary material for: Sniper: improved SNP discovery by multiply mapping deep sequenced reads
Source: Genome Biol. 2011 Jun 20;12(6):R55. doi: 10.1186/gb-2011-12-6-r55 (PMC3218843; doi:10.1186/gb-2011-12-6-r55)
Supplement: Additional file 12 — Figure S7 - genotyping accuracy for simulated data. Bar charts are shown reporting SNP identification accuracy on four synthetic genomic DNA templates (RPL, 2 × RPL +2%, 2 × RPL +5%, 2 × RPL +10%). Five unknown sample genomes were generated from each reference template by adding SNPs randomly to a proportion of 0.001. Read sets were sampled from each sample genome to one of nine coverage levels (4-fold, 10-fold, 25-fold, 32-fold, 50-fold, 75-fold, 100-fold, 150-fold, 200-fold). Read sets were independently aligned to their respective reference genome using ALL, UNI, or BEST maps with k = 1, 2, or 3 mismatches and genotyped using Sniper. Error bars show ± standard error of the mean over five replicates. [file gb-2011-12-6-r55-S12.PDF]

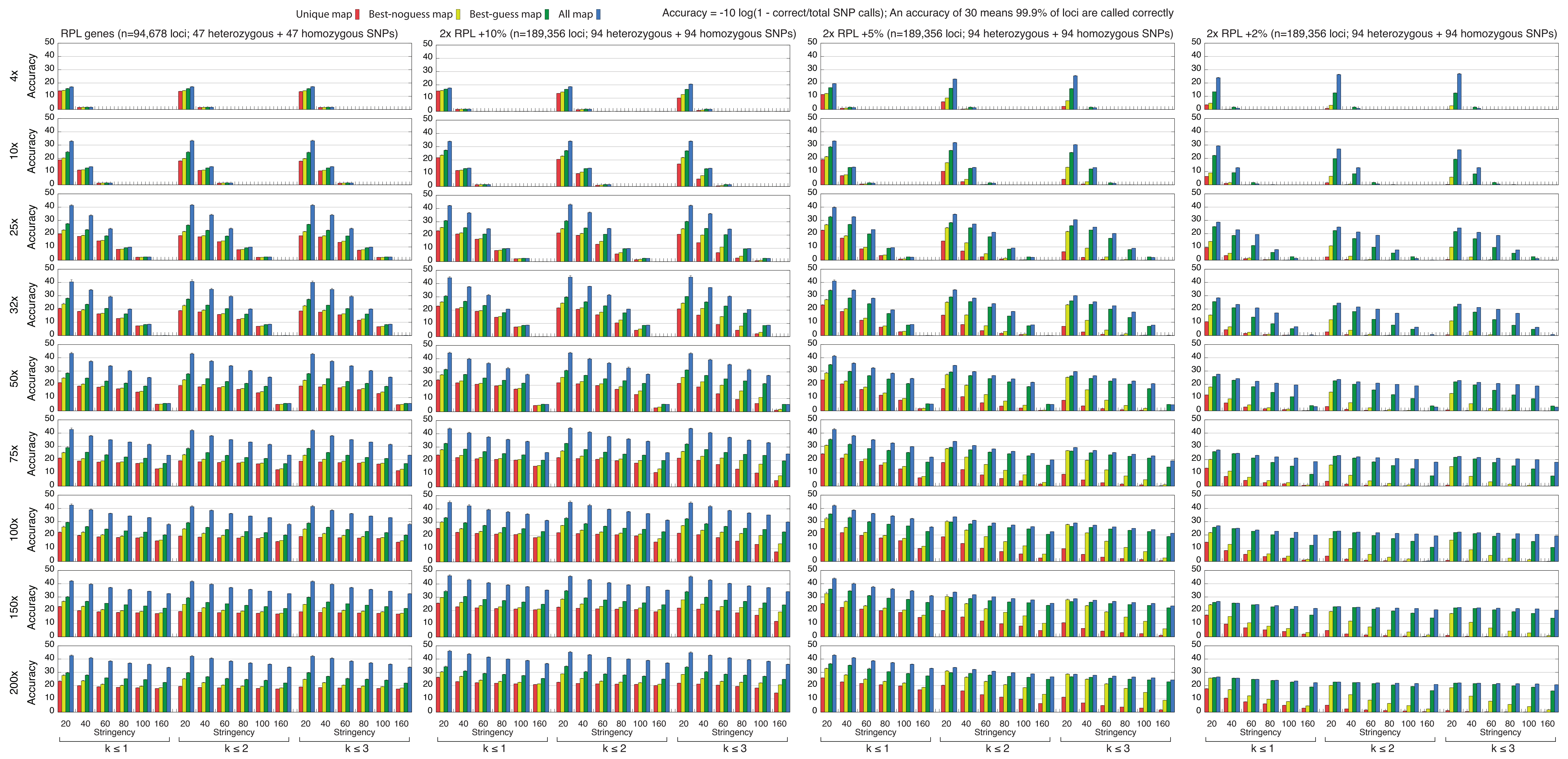
Unique map
Best-noguess map
Best-guess map
All map
Accuracy = -10 log(1 - correct/total SNP calls); An accuracy of 30 means 99.9% of loci are called correctly
RPL genes (n=94,678 loci; 47 heterozygous + 47 homozygous SNPs)
2x RPL +10% (n=189,356 loci; 94 heterozygous + 94 homozygous SNPs)
2x RPL +5% (n=189,356 loci; 94 heterozygous + 94 homozygous SNPs)
2x RPL +2% (n=189,356 loci; 94 heterozygous + 94 homozygous SNPs)
4x
10x
25x
32x
50x
75x
100x
150x
200x
Accuracy
20 40 60 80 100 160
Stringency
k ≤ 1
k ≤ 2
k ≤ 3
